# Reinvigoration of innate and adaptive immunity via therapeutic cellular vaccine for patients with AML

**DOI:** 10.1016/j.omto.2022.09.001

**Published:** 2022-09-23

**Authors:** Shin-ichiro Fujii, Toyotaka Kawamata, Kanako Shimizu, Jun Nakabayashi, Satoru Yamasaki, Tomonori Iyoda, Jun Shinga, Hiroshi Nakazato, An Sanpei, Masami Kawamura, Shogo Ueda, Jan Dörrie, Svetlana Mojsov, Madhav V. Dhodapkar, Michihiro Hidaka, Masanori Nojima, Fumitaka Nagamura, Shigemi Yoshida, Toshio Goto, Arinobu Tojo

**Affiliations:** 1 Laboratory for Immunotherapy, RIKEN Center for Integrative Medical Science (IMS), 1-7-22 Suehiro-cho, Tsurumi-ku, Yokohama, Kanagawa 230-0045, Japan; 2 Program for Drug Discovery and Medical Technology Platforms, RIKEN, 1-7-22 Suehiro-cho, Tsurumi-ku, Yokohama, Kanagawa 230-0045, Japan; 3 Department of Hematology/Oncology, The Institute of Medical Science, The University of Tokyo (IMSUT): Minato-ku, Tokyo 108-8639, Japan; 4 Department of Mathematics, Tokyo Medical and Dental University, Ichikawa, Chiba 272-0827, Japan; 5 Department of Dermatology, Universitätsklinikum Erlangen, 91052 Erlangen, Germany; 6 The Rockefeller University, New York, NY 10065, USA; 7 Department of Hematology and Oncology, Emory University School of Medicine, Atlanta, GA 30322, USA; 8 Department of Hematology/Oncology, National Hospital Organization Kumamoto Medical Center, Clinical Laboratory: Kumamoto, Kumamoto 860-0008, Japan; 9 Center for Translational Research, The Institute of Medical Science, The University of Tokyo (IMSUT), Minato-ku, Tokyo 108-8639, Japan

**Keywords:** AML, Dendritic Cell, Natural Killer T Cell, Natural Killer Cell, Cytotoxic T Cell, Memory T Cell, WT1, Immunotherapy, Artificial Adjuvant Vector Cell, Clinical Trial

## Abstract

Strategies integrating activation of innate and adaptive immunity against cancer are desired. We established a novel platform, Wilms’ tumor antigen 1 (WT1)-expressing artificial adjuvant vector cells (aAVC-WT1), linking invariant natural killer T (iNKT)-mediated dendritic cell activation to T cell immunity. Here, we report the first-in-human application of aAVC-WT1 in nine patients with relapsed and refractory acute myelogenous leukemia. No dose-limiting toxicities were observed, whereas activation of iNKT and/or NK cells was observed in all patients. Five patients experienced objective leukemic regression, which correlated with WT1-specific T cell responses. Paired single-cell RNA and T cell receptor (TCR) sequencing demonstrated effector CD8^+^ T cell clones in the bone marrow. Some bone marrow CD8^+^ T cells underwent transition from pre-existing precursor exhausted T cells to functional T cells or emerged as newly activated T cells, some of which were maintained long term. These demonstrate the feasibility and safety of aAVC-WT1 therapy and the capacity of this platform to activate both innate and adaptive immunity in humans.

## Graphical abstract

**Figure:**
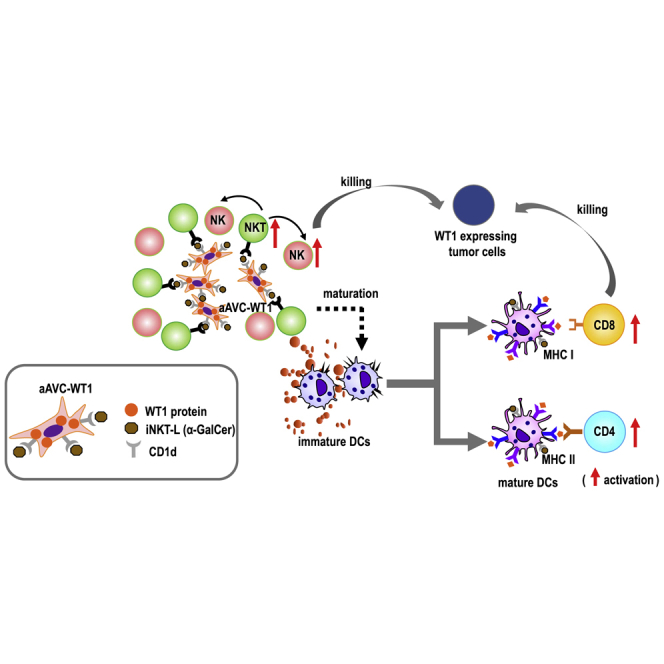

## Teaser

Based on the previous pre-clinical studies, we demonstrate NKT cell-licensed DC therapy integrating innate and adaptive immunity against relapse and refractory AML with safety in first-in-human study, and this can also induce the reinvigoration of pre-existing pre-exhausted T cells and the emergence of newly activated CD8^+^ T cells.

## Introduction

Generation of tumor-specific polyclonal T cells is desired to prevent the immune escape of tumor cells. Although several strategies incorporating short peptide-based single antigen cancer vaccines have been evaluated in clinical trials, tumor heterogeneity has limited the ability of these vaccines to stimulate robust immune responses. Contrary to expectations, protein-based vaccines also showed weak immunogenicity, due in part to insufficient capture and cross-presentation by antigen-presenting cells (APCs) to T cells.^1^ These observations highlight the importance of targeting the optimal APCs.^2^ Dendritic cells (DCs) are highly specialized APCs with central roles in immunity.^3^ Several types of *in situ* DC-targeting therapies, such as anti-DEC205 antibody-mediated targeting of surface receptor proteins on DCs^3^^,^^4^ and liposomal mRNA vaccines,^5^ have been studied. These studies have shown that selecting the appropriate adjuvants and antigens for targeting the optimal APCs is critical for generating efficacious therapeutic vaccines.

Invariant natural killer T (iNKT) cells are CD1d-restricted innate-like αβT cells expressing an invariant T cell receptor (TCR) (Vα14 in mice and Vα24 in human; hereafter, iNKT cells).^6^^,^^7^ Stimulation of iNKT cells by α-galactosylceramide (α-GalCer) induces the robust production of interferon gamma (IFN-γ), resulting in NK cell activation *in vivo* in mouse^8^^,^^9^ and human.^10^^,^^11^ Moreover, under optimal conditions, iNKT cells promote *in situ* maturation of DCs to an immunogenic state and exhibit adjuvant activity, leading to adaptive immunity.^9^^,^^12^^,^^13^^,^^14^^,^^15^^,^^16^^,^^17^ To optimize the iNKT cell/DC axis, we previously established the concept of an artificial adjuvant vector cell (aAVC) as a new type of DC-targeting cellular drug platform that incorporates iNKT-licensed DCs *in vivo*.^6^^,^^18^^,^^19^^,^^20^ aAVCs are allogenic cells containing tumor antigens with an α-GalCer-CD1d complex on their surface. After activation, innate iNKT/NK cells reject the aAVCs; however, the killed aAVCs are taken up by DCs *in situ*, thereby promoting several immunogenic features of DCs. In the lungs, liver, and spleen, the DCs that capture aAVCs undergo maturation via interaction with iNKT cells, brought about by CD40L/CD40 interactions and inflammatory cytokines (IFN-γ and tumor necrosis factor α [TNF-α]). These mature, immunogenic DCs then present tumor-associated antigens to T cells on both classes of major histocompatibility complex (MHC) *in situ*. Notably, aAVC treatment reduces the number of tumor metastases and eliminates grossly large tumors via activation of iNKT and NK cells and cytotoxic T lymphocytes (CTLs). Furthermore, aAVCs efficiently induce long-term memory T cells *in vivo*.^9^^,^^20^^,^^21^

In acute leukemia, impaired hematopoiesis and a decrease in the number of immune cells have been implicated in leukemic cell expansion in the bone marrow (BM). Recent studies in untreated, *de novo* acute myelogenous leukemia (AML) have demonstrated several alterations in immune cells. Some leukemic cells in AML express immune checkpoint molecules.^22^ Increased numbers of terminal exhausted CD8^+^ T cells, as well as impaired function of NK and T cells in the BM, have been described.^23^ Immunotherapy is an attractive approach to reprogram the status of BM in AML. However, whether immunotherapy can trigger reactivation of CD8^+^ T cells in the BM or generation of new CD8^+^ T cell clones in AML has not been demonstrated. Analysis of CD8^+^ T cells in the BM using single-cell RNA sequencing (scRNA-seq) approaches would provide insights into the tumor microenvironment in AML.

Wilms’ tumor antigen 1 (WT1) is overexpressed in several malignancies, including AML.^24^ Therefore, targeting WT1 may be helpful for immunotherapy.^25^ WT1-specific TCR-transfected adoptive T cell therapy has shown promising efficacy in AML.^26^ Vaccination with WT1-mRNA-loaded DCs led to induction of a WT1-specific response.^27^ Both immunotherapies were tested at complete remission (CR) state and led to the prevention or delay of relapse. Based on pre-clinical studies using aAVC-WT1,^20^ in this study, we performed the first-in-human study of aAVC-WT1-based cellular therapy in elderly patients with relapsed and refractory AML (RR-AML). The target population consisted of elderly patients with RR-AML (>60 years old), for whom newer therapies are urgently needed. To evaluate immune responses and changes in the abundances of specific immune cells, we tracked the responsiveness of iNKT and NK cells and several subsets of T cells responding to a broad array of epitopes on WT1-expressing leukemic cells. Furthermore, using high-dimensional profiling techniques, we studied the status, kinetics, and longevity of various T cell clones in the BM in patients with RR-AML by aAVC-WT1.

## Results

### Patient characteristics and treatment

aAVC-WT1 expresses the CD1d/α-GalCer complex on the surface and WT1 protein intracellularly (Figure S1A). To verify it, we usually assess the expression of CD1d and WT1 using flow cytometry and quantify WT1 protein using western blot analysis for aAVC-WT1. We observed the expression of CD1d on the cell surface and also of WT1 by intracellular staining using flow cytometry to assess the distribution of these molecules (Figure S1B). After quantifying WT1 by aAVC-WT1, we also examined the time kinetics of expression of both CD1d and WT1. We found that CD1d was stably expressed, whereas the expression of WT1 was decreased to the basal level after 24 h (Figure S1C). We previously demonstrated the mechanism and antitumor effect of aAVC-WT1 in a pre-clinical study using a murine model (Figure S1D).^20^

**Figure 1:**
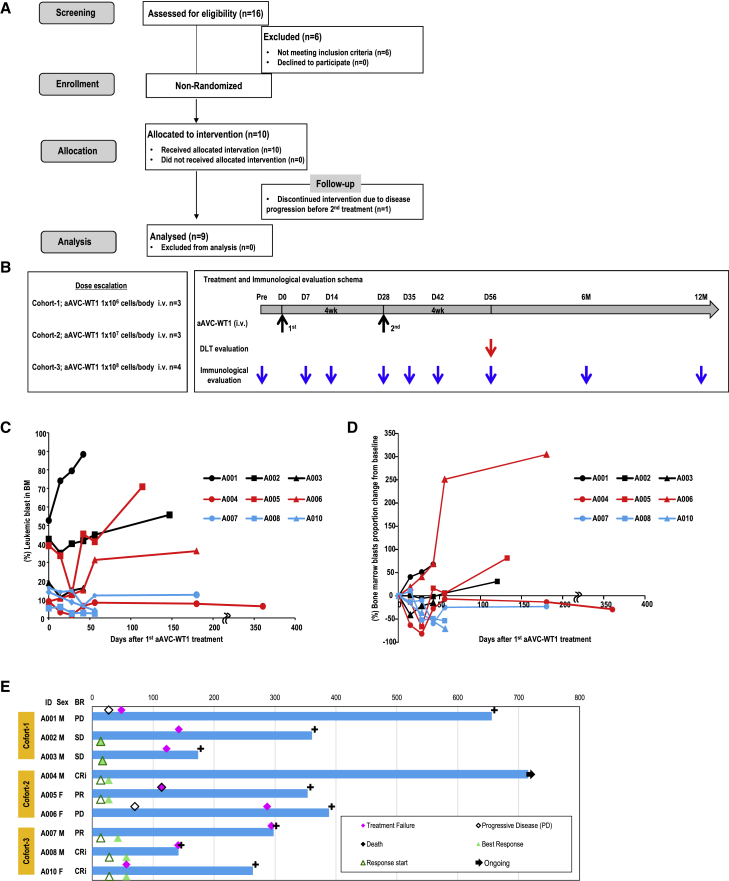
Clinical design and response (A) Flow diagram of the present study. (B) Patient allocation to treatment cohorts and schema. (C and D) The kinetics of bone marrow (BM) blast proportion (C) change from baseline ((BLAST_each_visit- BLAST_baseline)/BLAST_baseline) (D) in the patients who completed aAVC-WT1 treatment twice. The percentage of leukemic blasts (CD45^lo^) in the BM was evaluated using flow cytometry. BM blast proportion was determined by blast count from a BM smear in only A010. (E) Swimmer plots illustrating the response and outcome of patients with AML treated with aAVC-WT1.

After screening by assessing for eligibility, patients with RR-AML were enrolled, allocated for the therapy, underwent two cycles of aAVC-WT1, and then analyzed (Figure 1A; Table S1). The primary objective of this first-in-human phase I trial was to determine the maximum tolerated dose of this novel agent for further study, as well as to describe major toxic effects, including dose-limiting toxicity. The patients were divided into three cohorts of three patients each: cohort 1 (1 × 10^6^ cells per dose), cohort 2 (1 × 10^7^ cells per dose), and cohort 3 (1 × 10^8^ cells per dose) in the protocol (Figure 1B). After discontinuation of chemotherapy for at least 2 weeks before the therapy, aAVC-WT1 was administered at two cycles for each level intravenously in a prime-boost schedule at 1-month intervals (Figure 1B) based on the previous report for *ex vivo* DC therapy^28^ and pre-clinical studies using mice^18^^,^^20^ and canines (i.e., beagle dog, weighing approximately 10 kg).^19^ Concomitant treatments were not allowed except for the transfusion of red blood cells or platelets (A003, A004, A006, A007, A008, A009, and A010) or G-CSF (A008). A004 received stem cell transplantation, 2 years before aAVC therapy, as a past treatment; no other patient received such a treatment. A004 neither showed a flared-up episode of graft-versus-host disease nor received any other therapy. Nine of the ten enrolled patients with RR-AML underwent complete analysis per-protocol set (PPS) and were evaluated in our phase I dose-escalation trial of aAVC-WT1 (Figures 1A and 1B; Table 1). One patient (A009) underwent chemotherapy soon after the first administration of aAVC-WT1 due to disease progression and therefore did not complete the planned treatment. The median age was 71 years (range, 66–80 years), and the median number of previous treatment lines was 5. Dose escalation of aAVC-WT1 therapy proceeded as planned with no dose-limiting toxicities. We have summarized the observed adverse events in Tables 1 and 2. All grade 3 and 4 patients already had leukemia or therapy-related cytopenia before the immunotherapy: grade 2 (A002) or 3 (A006 and A008) lymphopenia, grade 2 (A006) or 3 (A005) neutropenia, and grade 2 (A002) leukopenia before the aAVC-WT1 therapy. Patient A006 experienced grade 3 anemia but required blood transfusion before aAVC-WT1 treatment. These alterations were transient; that is, these reverted within a week without any clinical symptoms. Besides these hematology-related adverse events, we detected asymptomatic transient CRP elevation in A006. These data demonstrate that aAVC-WT1 therapy was well tolerated.

**Table 1:** Clinical characteristics of patients

Cohort	Patient ID	F/M	Age	AML characteristics	WT1 expression	Disease status at screening (numbers of lines of previous treatment)	% blast in BM (FACS)	Hemogram at pre-treatment			Best of clinical response (ELN)	Adverse events	Outcome
								WBC (×10^2^/μL)	Hb (g/dL)	Plt (×10^4^/μL)			
1	A001	M	80	t(5; 12) (q35; q13) Flt3-ITD(−)	+	4th relapse (9 lines)	52.7	49.5	13.4	10.6	PD	–	D655 dead (progression)
1	A002	M	71	8 trisomy, Flt3-ITD(−)	+	1st relapse, resistance for rescue therapy (3 lines)	42.6	19.5	12.8	2.8	SD	leukopenia, neutropenia, lymphopenia, dyspepsia, arthralgia, erythema, sinus tachycardia,	D360 dead (progression)
1	A003	M	66	8 trisomy, Flt3-ITD(−)	+	1st relapse, resistance for rescue therapy (6 lines)	19	10.6	8.0	1.4	SD	rash, ear pain, eczema	D173 dead (progression)
2	A004	M	75	8 trisomy	+	1st relapse, resistance for rescue therapy (2 lines + CBT)	8.96	20.9	10.3	5.2	CRi	constipation, headache, hematuria, hepatic function abnormal	live (at day 715)
2	A005	F	75	7 monosomy, Flt3-ITD(−), NPM1(−)	+	2nd relapse (4 lines)	39.1	35.5	11.7	5.4	PR	anemia, rash, leukopenia	D353 dead (progression)
2	A006	F	70	Flt3-ITD(−)	+	resistance of induction therapy, resistance for rescue therapy (3 lines)	8.94	36.5	8.4	3.3	PD	oxygen saturation decrease, leukopenia, neutropenia, lymphopenia, C-reactive protein increased	D388 dead (infection)
3	A007	M	76	Flt3-ITD(−)	+	1st relapse resistance for rescue therapy (6 lines)	16.3	20.8	10.8	4.7	PR	–	D297 dead (infection)
3	A008	M	71	Flt3-ITD(−)	+	3rd relapse (9 lines)	5.37	86.5	8.2	2.2	CRi	lymphopenia, rash, myalgia	D141 dead (infection)
3	A009^a^	M	68	–	+	1se relapse, resistance for rescue therapy (2 lines)	16.6	27.0	8.9	3.9	PD	–	D304 dead (progression)
3	A010	F	68	Flt3-ITD(−), NPM1 (−), CEBPA(−)	+	2nd relapse, resistance for rescue therapy (6 lines)	14.0^a^	7.0	10.1	5.6	CRi	–	D263 dead (infection)

CBT; cord blood transplantation, PD; progression disease; SD; stable disease; CRi, complete remission with incomplete hematological recovery; PR, partial response.

a A009 discontinued intervention due to disease progression before second aAVC-WT1 treatment.

**Table 2:** Adverse events

Cohort	Adverse events	Patient per maximum CTCAE grade			
		1	2	3	4
1	neutrophil count decreased	0	0	0	1
	lymphocyte count decreased	0	0	1	0
	white blood cell decreased	0	0	1	0
	hyperemia	0	1	0	0
	sinus tachycardia	0	1	0	0
	dyspepsia	0	1	0	0
	eczema	0	1	0	0
	erythema	0	1	0	0
	rash	1	0	0	0
	purpura	1	0	0	0
	ear pain	1	0	0	0
	arthralgia	1	0	0	0
2	lymphocyte count decreased	0	0	1	0
	neutrophil count decreased	0	0	1	0
	anemia	0	0	1	0
	white blood cell decreased	0	2	0	0
	C-reactive protein increased	0	1	0	0
	constipation	1	0	0	0
	rash	1	0	0	0
	hepatic function abnormal	1	0	0	0
	hematuria	1	0	0	0
	oxygen saturation decreased	1	0	0	0
	headache	1	0	0	0
3	lymphocyte count decreased	0	0	1	0
	rash	1	0	0	0
	myalgia	1	0	0	0

All grade 3 or 4 patients already had leukemia or therapy-related cytopenia before the immunotherapy.

### Clinical response to aAVC-WT1 therapy

To objectively quantify and monitor the leukemic burden, we utilized an ordinal blast gating method, gating CD45^lo^ cells and validating some typical leukemic cell surface markers (CD34, CD33, CD38, CD13, and CD41) using flow cytometry (Figure S2).

We evaluated the clinical response of patients who had completed aAVC-WT1 treatments in PPS using European leukemiaNet (ELN) criteria,^29^ like in recent reports (Figures 1C–1E; Table 1).^30^^,^^31^^,^^32^ We initially evaluated the kinetics of the leukemic blast (Figure 1C). We also utilized spider plots and swimmer plots. The spider plots showed a change in the leukemic cell proportion from baseline in the BM (Figure 1D). As shown in Figure 1D, the number of leukemic cells reduced by >15% in patients A002, A003, A004, A005, A007, A008, and A010 after the therapy. Notably, we detected >50% reduction in the number of leukemic cells from baseline in five patients (A004, A005, A007, A008, and A010). Among these patients, patient A004 achieved CR with incomplete hematologic recovery (CRi) (from 8.96% to 1.62%), and patients A008 and A010 also achieved CRi (from 5.37% to 2.56% in A008 and from 14.0% to 4.0% in A010) after aAVC-WT1 therapy. After achieving CRi, patient A010 dropped out because of the desire to proceed to hematopoietic stem cell transplantation (HSCT) consolidation. We have summarized the relevant clinical events and the duration of survival in the nine patients using swimmer plots (Figure 1E). Contrary to the practice followed in some other chemotherapies or immunotherapies, we ceased all therapy after two doses of injection of aAVC-WT1. Therefore, the durability of clinical responses can be evaluated by the survival. Median overall survival (OS) (360 days) in this aAVC therapy compares favorably to previous reports in similar elderly patients with RR-AML. As an example, in prior studies, the median OS of these elderly patients with RR-AML aged >60 years was 4.5–4.9 months.^33^^,^^34^^,^^35^ Thus, aAVC-WT1 exhibited promising efficacy, with induction of disease stability and decreased leukemic cells in seven out of nine treated patients.

### Kinetics of iNKT and NK cells in aAVC-WT1-treated patients with RR-AML

**Figure 2:**
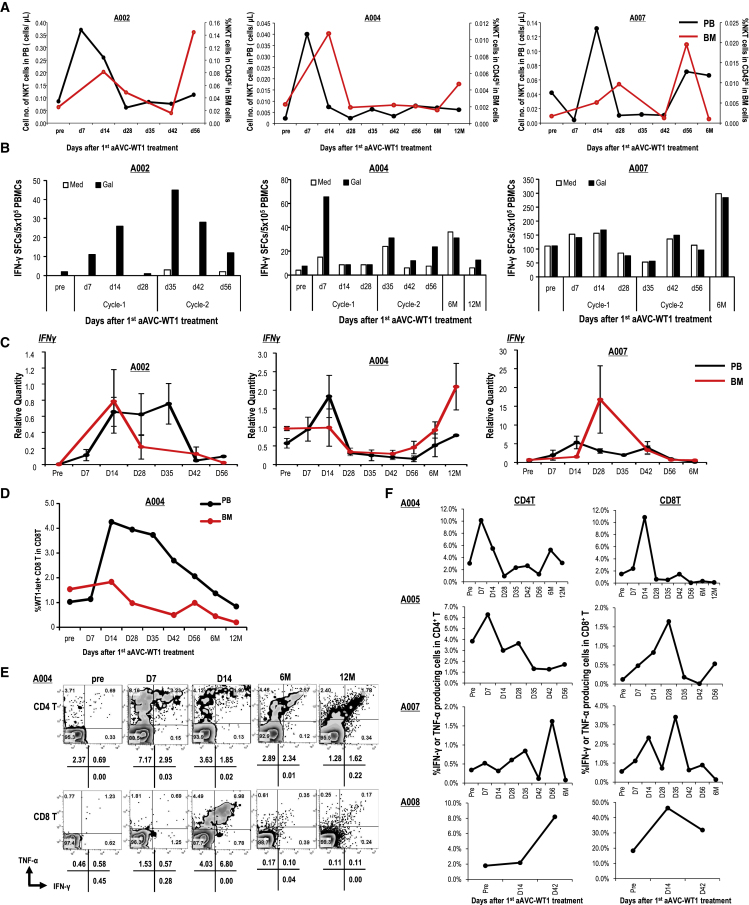
Innate and adaptive immune responses in aAVC-WT1-treated patients with RR-AML (A–C) iNKT and NK cell responses in patients A002, A004, and A007. (A) Frequency of iNKT cells (CD45^+^CD3^+^Vα24^+^Vβ11^+^) in CD3^+^ T cells in the PB (black) and BM (red) was analyzed using flow cytometry. (B) iNKT and NK cell responses in IFN-γ ELISPOT assays. (C) Expression of *IFNG* in NK cells sorted from the PB (black) and BM (red), as determined by qPCR (mean ± SEM, triplicates). (D–F) WT-1-specific T cell responses. (D) Kinetics of the frequency of WT1/HLA-A24:02 tetramer^+^CD8^+^ T cells in CD8^+^ T cells of primary PB (black) and BM (red) in patient A004.The tetramer-positive frequency was calculated by subtracting the frequency of the negative control (HIV peptide/HLA-A24:02 tetramer). (E) WT1-specific CD4^+^ T and CD8^+^ T cell responses in PB using the WT1 peptide library. Intracellular IFN-γ and TNF-α production by CD4^+^ T and CD8^+^ T cells was determined using flow cytometry. Representative flow cytometry data are shown for patient A004. Data on gating of CD3^+^CD4^+^ for CD4^+^ T cells (top) and CD3^+^CD8^+^ for CD8^+^ T cells (bottom) are shown. The numbers indicate the frequency of three subsets (TNF-α^+^IFN-γ^−^, TNF-α^−^IFN-γ^+^, and TNF-α^+^IFN-γ^+^) by subtracting the frequency of non-stimulated control. (F) The kinetics of WT1-specific IFN-γ- or TNF-α-producing CD4^+^ T and CD8^+^ T cells in A004, A005, A007, and A008 are summarized.

We focused on changes in the frequency or enhancement of the function of iNKT and NK cells in patients who had completed two aAVC-WT1 treatments because it indicated the activation of innate lymphocytes. The cell number in peripheral blood (PB) and the cell frequency in CD45^high^ cells in BM for each cell type (i.e., iNKT, NK, and T cells) at baseline and after treatment (best response) were assessed (Tables 3 and S2). Since patient A010 exhibited leukopenia prior to treatment, only the cell number of iNKT, NK, and T cells in PB could be analyzed (Tables 3 and S2). The results for three representative patients (A002, A004, and A007) in each cohort are shown in Figure 2A. The iNKT cells in the PB and BM increased for all three patients, and the ratio of iNKT cells in the pre-treatment state to that of the best response was increased by 1.5-fold in PB (4 of 9 patients) and BM (6 of 8 patients) (Table 3). These changes were more apparent in the BM than in the PB. Thus, we successfully observed increased iNKT cells in PB and/or BM in patients A001, A002, A003, A004, A007, and A008 (Table 3).

**Table 3:** iNKT and NK cell response.

Cohort	Pt. no.	Sample	iNKT post^a^/pre (fold)	NK post^a^/pre (fold)
1	A001	PB∗∗	0.11/0.06 (1.83)	162.21/56.97 (2.90)
	A002		0.37/0.09 (428)	29.75/5.63 (5.28)
	A003		0.02/0.04 (0.42)	52.7/10.86 (5.77)
	means ± SEM (fold)		2.18 ± 0.92	4.65 ± 0.72
	A001	BM∗∗∗	0.015%/0.007% (2.28)	13.8%/20.3% (0.68)
	A002		0.145%/0.025% (5.71)	17.5%/10.9% (1.61)
	A003		0.014%/0.004% (3.48)	13.2%/4.93% (2.68)
	means ± SEM (fold)		3.82 ± 0.82	1.65 ± 0.47
2	A004	PB∗∗	0.08/0.01 (12.59)	145.42/61.16 (2.38)
	A005		0.20/0.22 (0.89)	140.58/132.16 (1.06)
	A006		0.03/0.05 (0.49)	20.60/3.80 (5.42)
	means ± SEM (fold)		2.24 ± 1.02	2.95 ± 1.05
	A004	BM∗∗∗	0.011%/0.002% (4.71)	29.2%/25.6% (1.14)
	A005		0.020%/0.016% (1.28)	12.8%/8.58% (1.49)
	A006		0.006%/0.008% (0.73)	4.64%/0.96% (4.83)
	means ± SEM (fold)		2.18 ± 0.92	2.49 ± 0.96
3	A007	PB∗∗	0.13/0.04 (3.11)	372.20/373.63 (1.00)
	A008		0.20/0.43 (0.47)	121.27/384.06 (0.32)
	A010		0.02/0.06 (0.25)	71.55/30.69 (2.33)
	means ± SEM (fold)		1.28 ± 0.75	1.21 ± 0.48
	A007	BM∗∗∗	0.02%/0.002% (11.26)	30.6%/21.3% (1.44)
	A008		0.076%/0.017% (4.38)	35.9%/12.1% (2.97)
	A010		ND	ND
	means (fold)		7.82	2.20

∗∗ (cells/μL) in PB; ∗∗∗ relative frequency (%) in BM CD45hi cells. ND, not done.

a Best response in all time course.

NK cells can be activated by the adjuvant effect of activated iNKT cells.^8^ The ratio of NK cells in the pre-treatment state to that at the best response was increased by 1.5-fold in the PB (5 of 9 patients) and BM (4 of 8 patients) (Table 3). Consequently, we successfully showed that the frequency of NK cells in PB and/or BM increased in patients A001, A002, A003, A004, A006, A008, and A010 (Table 3). Thus, we observed an increase in the frequency of iNKT and/or NK cells in PB and/or BM in all the patients except A005 (Figure 2A; Table 3).

To study the functional enhancement of iNKT/NK cells, we focused on the increase in IFN-γ production, measured using IFN-γ ELISPOT assay for iNKT and NK cells or using real-time PCR for IFN-γ of NK cells. The number of ligand-dependent IFN-γ spot-forming cells (SFCs) was increased in patients A002 and A004 after aAVC-WT1 treatment, suggesting that these are the ligand-specific iNKT cell-mediated responses (Figure 2B). However, ligand-independent IFN-γ production in patient A007 suggested that iNKT and/or NK cells were activated. Quantitative real-time PCR analysis showed elevation of *IFNG* expression in NK cells sorted from the PB and BM, demonstrating NK cell activation in patients A001, A002, A003, A004, A005, and A007 (Figures 2C and S3A). The enhanced function of the innate lymphocytes was shown using IFN-γ ELISPOT assay or real-time PCR for IFN-γ of NK cells (A001, A002, A003, A004, A005, and A007). Taken together, we detected an increased frequency and/or increased IFN-γ production of iNKT or NK cells in all patients. Notably, iNKT and NK cells seemed dysfunctional or remained naive (dormant) at pre-treatment, but both were reactivated by aAVC-WT1 therapy.

### Broad response of T cells for the multiple epitope-WT1 antigen

We screened HLA-A24^+^ patients to analyze HLA-A24:02/WT1 tetramer^+^CD8^+^ T cells in this study. We assessed the frequency of antigen-specific CD8^+^ T cell responses to class I epitopes from WT1 by direct HLA-A24:02/WT1 tetramer in four patients (A001, A003, A004, and A005; Figures 2D, S3C, and S3D). To assess the background of this assay in addition to using HIV tetramer in staining, we analyzed the samples from 12 HLA-A24^+^ healthy donors (HDs) and 10 HLA-A24^+^ patients with AML who had not been treated with aAVC-WT1 (Figure S3B). Further, we analyzed two HLA-A24^−^ patients treated with aAVC-WT1 (A007 and A008). We calculated the frequency of HLA-A24:02/WT1 tetramer in CD8^+^ T cells by subtracting the frequency of the negative control (HIV peptide/HLA-A24:02 tetramer). The mean ± 2 standard deviation for the HLA-A24^+^ healthy individuals and HLA-A24^−^ patients with AML was 0.081 ± 0.167. Therefore, we determined the threshold of HLA-A24:02/WT1 tetramers as 0.248% (Figure S3C). For data presented in Figure S3B, there was no statistical difference in the percentage of WT1 tetramer^+^CD8^+^ T cells between HD and patients with AML. The number of WT1 tetramer^+^CD8^+^ T cells was increased in the PB and/or BM after aAVC-WT1 therapy in three of four patients (A001, A004, and A005) (Figures 2D and S3D). The peak for the increased frequency of WT1 tetramer^+^CD8^+^ T cells was observed at one of the three time points (day 14, 35, or 42). These were 1 or 2 weeks after the administration of aAVC-WT1. It is notable that HEK293 cells, which we use as vector cells for aAVC-WT1, are positive for HLA-A∗02:01 but not HLA-A∗24:02. Therefore, aAVC-WT1 cannot directly present WT1 antigen to HLA-A24:02/WT1 tet^+^CD8^+^T cells, supporting DC-mediated cross-presentation of WT1 antigen *in vivo* in aAVC-WT1-treated patients, consistent with pre-clinical studies.^20^

Next, we focused on the adaptive immunity in four patients (A004, A005, A007, and A008), who showed a 50% or more reduction in the number of leukemic cells in the BM following aAVC-WT1 therapy. The aAVC system expresses the whole target antigen and can enhance CD4^+^ and CD8^+^ T cell responses in an antigen-specific manner.^18^^,^^19^^,^^20^^,^^35^ The antigen-specific T cell responses relevant for the whole WT1 protein were assessed by culturing PB mononuclear cells (PBMCs) with an overlapping 15-mer peptide library. Furthermore, to detect the diversity of the T cell-responding epitopes of WT1, we focused on the T cell response in patients A004 and A007, who were in a progression-free (or stable) state for an extended period. To assess their WT1-derived broad and multifunctional T cell response, we utilized 10 subpool mixtures, including 12–13 peptides (Figures 2E, 2F, S4, and S5; Table S3). In patient A004, CD4^+^ T and CD8^+^ T cell responses to the total peptide library were enhanced 7 and 14 days after the first aAVC-WT1 treatment, respectively. Both CD4^+^ and CD8^+^ T cell responses lasted for 6–12 months. To verify the response epitopes, CD4^+^ and CD8^+^ T cells were assessed after restimulation with subpooled peptides; IFN-γ^+^TNF-α^+^CD4^+^ T cells in the pretreated state mainly responded to subpools 3, 6, and 7, with robust responses extending to subpools 1, 3, and 5–8 after aAVC-WT1 treatment (Figures S5A and S5B). Interestingly, we detected CD4^+^ T cells responding to 1, 3, and 5 subpools, even after 12 months (Figures S5A and S5B). IFN-γ^+^TNF-α^−^ and IFN-γ^−^TNF-α^+^, but not IFN-γ^+^TNF-α^+^CD8^+^, T cells responded to the peptide libraries in the pre-treatment stage; however, on days 7 and 14, IFN-γ^+^TNF-α^+^CD8^+^ T cells responding to almost all the subpools were detected, and those responding to subpools 3 and 7 persisted for 12 months after the aAVC-WT1 therapy (Figures S5A and S5B). In patient A005, antigen-specific CD4^+^ T cell responses were detected at the pre-treatment stage and were enhanced on day 7 (Figures 2F and S4). In addition, antigen-responsive CD8^+^ T cells that produced IFN-γ expanded well on day 28 (Figures 2F and S4). In patient A007, total peptide library-responsive CD4^+^ T cells gradually increased after the second aAVC-WT1 administration (Figure 2F). In contrast, total peptide library-responsive TNF-α-producing CD8^+^ T cells expanded well on day 14 after the first dose and were boosted after the second dose through day 35 (Figures 2F, S5C, and S5D). IFN-γ^+^TNF-α^+^CD8^+^ T cells were widely detected in all subgroups except in subpool 9 on day 14, and such effective CD8^+^ T cells persisted for 6 months (Figures S5C and S5D). In patient A008, we predominantly detected library-responsive IFN-γ^+^TNF-α^+^CD8^+^ T cells at pre-treatment, and these CD8^+^ T cells expanded on day 14, persisting through day 42 (Figure S4).

Hence, effector CD8^+^ T cell frequencies increased after the aAVC-WT1 treatment in all four patients, although most patients had a low frequency of WT1-responding pre-existing T cells at the pre-treatment stage (Figure 2F). Above all, in patients A004 and A007, a variety of T cell responses to several subpooled peptide libraries were generated, indicating induction of broad and diverse antigen-specific T cells. These T cell clones acquired the ability to produce multiple cytokines, such as IFN-γ and TNF-α. Even though the frequency of pooled peptide-responsive CD8^+^ T cells was low, the population responding to the subpeptide pool was detected for an extended period, indicating the existence of multifunctional memory T cells. Accordingly, these findings suggested that the WT1-responsive pre-existing T cell population expanded and was reactivated or that new epitope-responsive T cell clones were generated after aAVC-WT1 therapy. When we assessed the timing between the reduction in leukemic blasts and CD4^+^ T or CD8^+^ T cell response to the peptide libraries, we found that the best tumor cell reduction occurred at the same time or a little after the peak of CD8^+^ T cell response (best tumor reduction was at days 28, 28, 42, and 28), whereas library-responsive T cell peak was at days 14, 28, 35, and 14 in A004, A005, A007, and A008, respectively. This indicated that WT1 antigen-specific CD8^+^ T cells correlated with tumor reduction.

### Profiling of T cells infiltrated in the leukemic BM and PB of a patient treated with aAVC-WT1

**Figure 3:**
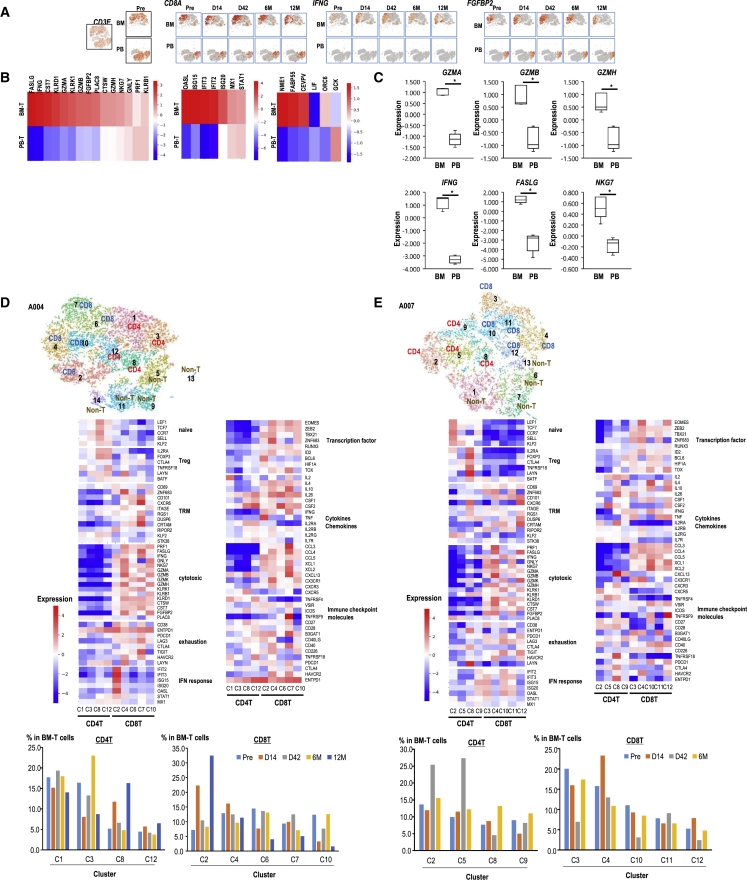
Differences in gene expression in T cells from PB and BM, and T cell heterogeneity in BM (A) *CD8A*, *FGFBP2*, and *IFNG* expression in BM T cells (top) and PB T cells (bottom) from patient A004, as shown in tSNE plots. (B) Heatmap of gene-expression signatures (cytotoxic, IFN response, proliferation) in BM-T cells (top) and PB-T cells (bottom) from patient A004. Average expression levels through all time points are shown in the heatmap. The color density indicates the average expression. (C) Expression levels of 6 genes (*GZMA*, *GZMB*, *GZMH*, *FASL*, *INFG*, *NKG7*) are compared between BM and PB T cells. Samples were obtained from BM and PB at five time points (pre, days 14 and 42, and months 6 and 12). (mean ± SEM) *∗*p *<* 0.05, Mann-Whitney *U* test. (D and E) Characterization of BM-CD4^+^ T and CD8^+^ T clusters in patients A004 (D) and A007 (E). The tSNE projection of single T cells from patient A004 showed the formation of 14 clusters, including five for CD8^+^ T, four for CD4^+^ T, and four for non-T cells (D), whereas the tSNE projection of single T cells from A007 showed the formation of 13 clusters, including five for CD8^+^ T, four for CD4^+^ T, and four for non-T cells (E). Each dot corresponds to a single cell, colored according to the cell cluster (top). Average expression through all time points of selected T cell function-associated genes in each cell cluster (middle). Kinetics of the frequency of each cluster (bottom).

scRNA-seq was used to evaluate the gene signatures of T cells in the BM (BM-T) and PB (PB-T) of patient A004 because substantial and long-term protection against an increase in the number of leukemic cell growth was observed. The different components were mapped by t-distributed stochastic neighbor embedding (tSNE) analysis (Figure 3A). To compare the expression data between BM- and PB-T cells, all the data from different time points were integrated, and the average expression levels were assessed. The levels of effector markers, such as cytotoxic scores, proliferation markers, and IFN-responsive markers, were increased in the BM but not in the PB (Figure 3B). In particular, six tumor cytotoxicity-related molecules (*GZMA*, *GZMB*, *GZMH*, *IFNG*, *FASLG*, and *NKG7*) from T cells had significantly higher levels in the BM than in PB (Figure 3C).

### Unbiased definition of CD8^+^ T cell states and their association with aAVC-WT1 therapy

In addition to reducing the number of leukemic cells, WT1-specific T cell response was elicited in four patients (A004, A005, A007, and A008) after the aAVC-WT1 therapy. We characterized the intrinsic T cell heterogeneity in each patient by measuring gene expression using single-cell transcriptomics. We integrated all the data at multiple time points of each patient individually and created tSNE plots to segment the clusters (Figures 3D, 3E, S6B, and S6C, top, in all cases). After calculating the average gene expression in each cluster for all the time points through the course of time, a heatmap of gene-expression signatures in each cluster was generated (Figure 3D, 3E, S6B, and S6C, middle, in all cases). When they were cross-labeled with those of a previous report,^36^ these were profiled as cluster-specific gene expression (Figure S6A). Finally, we demonstrated the kinetics of each cluster, which was determined as above, at each time point before and after the aAVC-WT1 therapy (Figure 3D, 3E, S6B, and S6C, bottom, in all cases). From this cross-labeled analysis, T cells were characterized and classified. FGFBP2-type cytotoxic effector T cells showed a cytotoxic score-related gene profile (*CX3CR1*, *PRF1*, *GNLY*, *NKG7*, *GZMB*, *KLRD1*, and *FGFBP2*). In contrast, IFN-responsive gene type effector T cells expressed IFN-responsive genes (*IFIT2*, *IFIT3*, *OASL*, and *ISG15*). Both effector T (T_EFF_) cells are often expressed by T cell activation cytokine genes, including *IFNG* and *TNF*. Exhausted T cells expressed *PDCD1*, *HAVCR2*, *LAYN*, *TIGIT*, *LAG3*, and *CD39* as gene signatures of chronic activation or T cell dysfunction. Recent reports have shown a remarkable phenotypic diversity in exhausted T cells within intratumoral T cell pools.^37^^,^^38^ These T cells, existing in a dysfunctional state, are characterized by high expression of *GZMK* and intermediate expression of *PDCD1* and *LAG3* and are referred to as “transitional T cells,” “pre-exhausted T cells,” or “T_PEX_ cells.” Although tissue-resident memory T (T_RM_) cells generally express typical markers (e.g., *CRTAM*, *RGS1*, *DUSP6*, *CD69*, *CXCR6*, and *TNFRSF9*), T_RM_ cells in aAVC-WT1-treated patients with AML expressed T_RM_ signatures and typical exhaustion markers.

Based on these criteria, we annotated different clusters in single-cell data and evaluated the kinetics of characterized clusters before and after the aAVC-WT1 therapy. Using cluster characterization, we evaluated the kinetics of each cluster during the course of time in individual patients. We detected five clusters of CD8^+^ T cells (C2, C4, C6, C7, and C10) and 4 clusters of CD4^+^ T cells (C1, C3, C8, and C12) in patient A004 (Figure 3D). After treatment, the levels of C2, C4, and C7 in CD8^+^ T cells were increased, whereas those of C6 and C10 were decreased. CD8-C2 expressed high levels of IFN-related genes and several cytotoxic score-related genes, which are indicators of IFN-T_EFF_ cells (IFN-related gene-effector T cells). CD8-C4 and -C10 expressed *FGFBP2* and other cytotoxic markers and some exhaustion markers, indicating that these were FGFBP2-T_EFF_ cells (FGFBP2-type cytotoxic effector T cells). CD8-C6 expressed *GZMK* as well as some exhaustion markers, suggestive of T_PEX_ cells. Although CD8-C7 expressed cytotoxic markers, this cluster simultaneously expressed exhaustion markers and *TOX*. In addition, CD8-C7 expressed high levels of T_RM_-positive markers and low levels of typical negative T_RM_ markers (STK38, KLF2, and RIPOR2), indicating the presence of pre-exhausted T_RM_ cells. It was similar to those in the reference clusters of CD8-C3-GZMK, CD8-C5-ZNF683 T_RM_, and CD8-C6-LAYN in solid tumors (Figure S6A).^36^ C8 among CD4^+^ T cells increased after therapy. Both CD4-C8 and -C12 expressed *IL2RA* and *FOXP3*, which are indicative of regulatory T cell (Treg) clusters. CD4-C3 expressed naive T (TN) markers, suggestive of TN cells. C1 was an unclassified cluster. Thus, two types of major effector CD8^+^ T cell populations (C2 and C4) and simultaneously pre-exhausted T_RM_ cells (C7) were increased. Thus, an effector-dominant immune response was elicited by the aAVC-WT1 therapy in patient A004.

In patient A007, we detected five clusters of CD8^+^ T cells (C3, C4, C10, C11, and C12) and four clusters of CD4^+^ T cells (C2, C5, C8, and C9; Figure 3E). C4 and C12 in CD8^+^ T cells and C2 and C5 in CD4^+^ T cells were increased after therapy, whereas C3 and C10 in CD8^+^ T cells and C8 and C9 in CD4^+^ T cells were decreased. CD8-C3 and -C11 were both IFN-related genes and FGFBP2 cytotoxic score-related genes expressing effector cells. Notably, CD8-C10 expressed *GZMK* and exhausted T cell-related molecules, suggestive of T_PEX_ cells. CD8-C12 expressed high levels of T_RM_ markers, with several cytotoxicity-related markers and exhaustion-related markers, suggestive of pre-exhausted T_RM_ cells. CD8-C4 expressed *FGFBP2* and many types of cytotoxicity-related markers, suggesting that CD8-C4 cells were FGFBP2-type T_EFF_. In CD4^+^ T cells, CD4-C8 expressed Treg markers, suggestive of Tregs. Additionally, CD4-C2 expressed TN markers, whereas CD4-C5 cells were defined as undifferentiated. CD4-C9 expressed *BCL6* and *CXCL13*, indicating a follicular helper T cell (T_FH_)-like population.^39^ Thus, two types of major CD8^+^ T cell populations, that is, CD8^+^ T_EFF_ cells (C4) and pre-exhausted T_RM_ cells (C12), were increased, whereas effector CD8^+^ T cells (C3) and T_PEX_ cells (C10) were simultaneously decreased. Both Tregs and T_FH_-like T cells were transiently decreased. Thus, an effector-type response was elicited by the aAVC-WT1 therapy in A007.

We also studied patients A005 and A008. We detected five clusters of CD8^+^ T cells (C4, C5, C6, C7, and C8) and two clusters of CD4^+^ T cells (C1 and C3) in patient A005 (Figure S6B). CD8-C5 and -C7 expressed *FGFBP2* with cytotoxic markers, such as FGFBP2-type T_EFF_ cells. CD8-C5, CD8-C7, CD4-C1 (TN), and CD4-C3 (Tregs) were increased. In contrast, pre-exhausted T_RM_ cells (CD8-C4) and IFN-related effector CD8^+^ T cells (CD8-C8) were decreased. In patient A008, we detected four clusters of CD8^+^ T cells (C6, C7, C8, and C10) and four clusters of CD4^+^ T cells (C1, C2, C11, and C13) (Figure S6C). Two FGFBP2-type T_EFF_ (CD8-C6 and -C10), T_PEX_ (CD8-C8), unclassified (CD4-C1), and naive (CD4-C2) clusters were increased. In contrast, pre-exhausted T_RM_ cells (CD8-C7), T_CM_ cells (CD4-C11), and Tregs (C13) were decreased. Thus, an effector-dominant type immune response was elicited by the aAVC-WT1 therapy in A005 and A008, indicating an effector cell-dominant immune response. Overall, although therapy-related immunological patterns differed in each patient, an effector CD8^+^ T cell-dominant immune response was elicited in five patients by aAVC-WT1 therapy.

### Relationship of peripheral and intra-BM CD8^+^ T cell clones after aAVC-WT1 therapy

**Figure 4:**
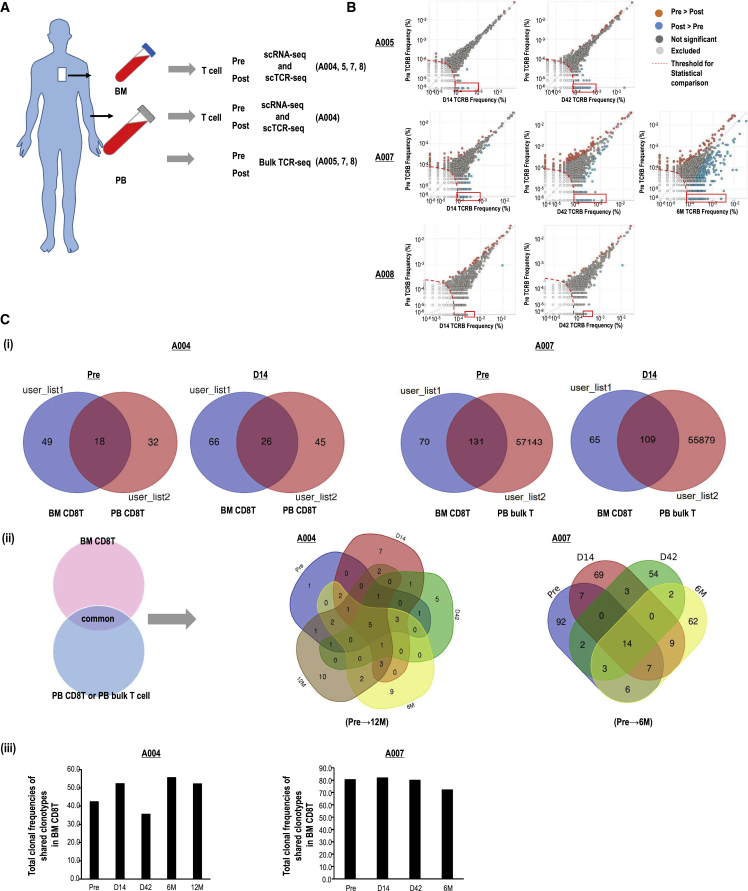
TCR repertoire analysis in PB and BM (A) Assay protocol for scRNA-seq, scTCR-seq, and bulk TCR-seq. (B) Scatterplots comparing T cell receptor Vβ (TRB) clone frequencies pre- and post-treatment using immunoSEQ platform, as measured by bulk TCR-seq for A005, A007, and A008. Significantly expanded clonotypes post-therapy are shown in orange. Clonotypes that show a significant reduction post-therapy are shown in blue. Red open squares indicate new emerging T cell clonotypes after the therapy. (C) Overlap between TRB clones in PB and BM T cells detected by scTCR-seq (A004) and bulk TCR-seq (A007) for PB and scTCR-seq for TILs. (i and ii) Venn diagram showing the sharing of TCR clonotypes in the BM and PB (A004 and A007) (i), and kinetics of shared T cell clonotypes from pre-treatment stage to day 42 or to 12 months post-treatment (A004) or those from pre-treatment stage to 6 months post-treatment (A007). (iii) The total clonal frequencies of shared TCR clones in BM-CD8^+^ T cells in A004 and A007.

We have thus far demonstrated that effector-dominant CD8^+^ T cells infiltrated the BM in addition to the increase in WT1-specific T cell response in PBMCs in aAVC-WT1-treated patients. We then investigated how the expansion of pre-existing or new clones contributed as effector T cells in both the BM and PB at several time points. For these purposes, we utilized single-cell TCR sequences of the BM and PB of patient A004. Alternatively, we assessed total CDR3β sequences of T cells in the PB by bulk TCR-seq and the BM by single-cell deep TCR-seq for patients A005, A007, and A008 (Figure 4A). To determine the number of clonotypes and the significant changes in PBMCs of each patient following aAVC-WT1, the two-sided binomial test was used (A005, A007, and A008; Figure 4B). Most clonotypes did not differ between pre- and post-aAVC-WT1 therapy in three patients. Several pre-existing clonotypes were expanded on day 42 and after 6 months in A007. New clones were detected post-therapy in A005 and A007 but not in A008. These results suggested that kinetics of CD8^+^ T cells needed to be followed at the clone level in individual patients after the treatment. We verified the existence of common CD8^+^ T cell clones in both BMMCs and PBMCs as shared clones (Figures 4Ci and S7A). After calculating the number of shared T cell clones, we evaluated the clonal frequencies and the kinetics of shared CD8^+^ T cell clones occupied in CD8^+^ T cells in the BM (Figures 4Ciii and S7B). The clonal frequencies of total shared clones accumulated in the BM CD8^+^ T cells did not alter before and after treatment but remained relatively high, that is, 30%–50% of CD8^+^ T cells in A004 and 70%–90% of CD8^+^ T cells in A005, A007, and A008 (Figures 4Ciii and S7B). In contrast, the shared T cell clonotypes comprised transient, medium-lived, and long-lived clones (Figure 4Cii). As discussed above, we detected not only the increase in the WT1-specific T cell response in PBMCs but also the FGFBP2-type effector CD8^+^ T cell populations in the BM in all the responders. These findings implied that several T cell clones in the BM underwent a functional change from pre-existing CD8 T_PEX_ cells and/or were newly formed, resulting in the development of WT1-specific effector FGFBP2 T cells after therapy.

### Functional alteration of CD8^+^ T_PEX_ cells following aAVC-WT1 therapy

**Figure 5:**
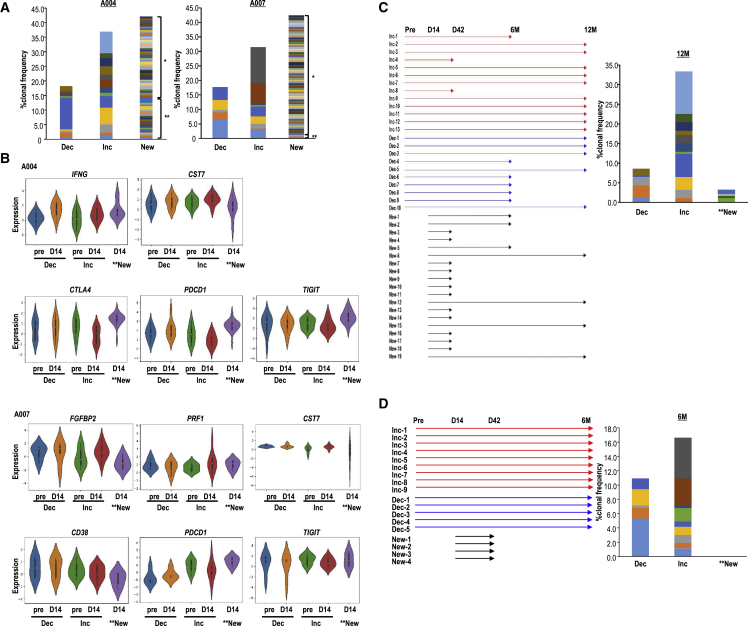
Kinetics of CD8^+^ T clonotypes in BM (A–D) Increased clones (as “Inc”) and decreased clones (as “Dec”) are defined in the clone frequency between the pre-treatment stage to day 14 or 42 post-treatment. Undetectable at pre-treatment but detectable at day 14 and/or 42 is defined as “new” (∗ indicates the transient clones only at day 14, whereas ∗∗ indicates stable clones remaining from day 14 to 42). (A) Clone frequencies of Inc, Dec, and “new” in BM-CD8^+^ T cells on day 14 in patients A004 and A007. (B) Expression of a given gene in the Inc, Dec, and ∗∗new groups at pre-treatment and on day 14 in patients A004 and A007. (C and D) Longevity (left) and clonal frequencies (right) of representative clones in the Inc, Dec, and ∗∗new groups at 12 months post-treatment for A004 (C) and 6 months post-treatment for A007 (D).

Next, we focused on the lifespan and function of preexisting clones or novel emerging clones individually. The kinetics of the CD8^+^ T cell clonotypes indicated that the major CD8^+^ T cell population could be classified into “increased (Inc)” or “decreased (Dec)” and “new” clones (Figures 5A and S8A). The new clones were separated into a transient subtype (disappearing by day 42) and a stable subtype (persisting on day 42).

Interestingly, the kinetics of expression profiles showed that Inc clones in A004, A007, and A008 exhibited upregulation of cytotoxic markers and downregulation of exhaustion markers (Figures 5B and S8B). New clones, defined as newly emerging T cell clones on days 14 and 42, expressed *IFNG* in A004 and *PRF1*and *CST7* in A007 (Figure 5B). Additionally, these clones showed high expression of immune checkpoint markers, such as *CTLA4*, *PDCD1*, and *TIGIT*, in A004 and A007. Despite being traditionally considered exhaustion T cell markers,^40^ several reports have shown that *PD-1*, *LAG-3*, and *TIM-3* are expressed preferentially in antigen-experienced TILs.^41^^,^^42^ Thus, the newT cell clones may have been activated or reactivated recently. DecT cell clones mostly expressed low levels of cytotoxic markers but high levels of exhaustion markers, suggesting that some clones were terminal exhausted T cells. *TCF7* plays a central role in conventional memory T cells and T_PEX_ cells.^37^ *TCF7* expression at pre-treatment was relatively higher in Inc clones than in Dec clones but was downregulated upon activation (Figures S8C and S8D). Thus, after the therapy, not only newly emergent T cell clones but also T cell clones derived from pre-existing T cells became more effective and reinvigorated.

Next, we assessed whether such reinvigorated T cell clones or new clones with effector functions could become long-term memory T cells or long-lasting T_PEX_ cells by tracing individual clones (Figures 5D and 5E). In patient A004, long-lasting T cell clones were mainly derived from Inc clones (Figure 5D). Additionally, in patient A007, the long-lasting T cell clones were derived from both Inc and Dec clones. However, new clones in patient A007 were relatively short-term effectors (Figure 5D). As reported earlier, exhausted T cells are separated into T_PEX_ and terminal exhausted cells.^37^^,^^38^ Among the Dec T cells, stable, long-lasting T cells might be derived from T_PEX_ cells. Thus, the aAVC-WT1 therapy enhanced invigorated T_PEX_ cells, with the stable cytotoxic markers exhibiting long-term survival.

## Discussion

In this study, we evaluated the antitumor efficacy of aAVC-WT1 in an aggressively growing mouse leukemia model and a first-in-human trial for elderly patients with RR-AML, who are known to have poor prognosis compared with younger counterparts.^33^^,^^34^^,^^35^ After intravenous administration, aAVC-WT1 enabled body-wide delivery of WT1 to APCs in various lymphoid tissues and concomitant initiation of the NKT-licensed DC-driven immunostimulatory program. Therefore, we specifically focused on this challenging target population. We simultaneously assessed the innate and adaptive immune responses. First, we showed that iNKT and NK cells were dysfunctional and produced only low levels of IFN-γ in steady-state RR-AML. Our results demonstrated dramatic activation of iNKT and NK cells in the BM of RR-AML after the aAVC-WT1 therapy, suggesting the reinvigoration of innate immunity. We also detected an increase in WT1-specific HLA-A24 tetramer^+^ T cells, indicating uptake and cross-presentation of the vaccine cells aAVC-WT1, which are HLA-A24^−^. Vaccine-induced T cell response included several subpools of a WT1 peptide library, indicating a broad and multifunctional T cell response during the effector phase. Vaccine-induced CTLs changed from TNF-α single-positive to IFN-γ^+^TNF-α^+^ T cells, indicating that reinvigorated T cells are prominent effector cells. We also observed long-lasting, reinvigorated T_PEX_ cells as functional T_PEX_ cells. To confirm whether T cell responses from the activation phase moved to the memory phase at a clonal level, we tracked T cell clones using scRNA and TCR repertoire analysis. Tracing of cell clones revealed both reinvigoration of pre-existing clones in the BM and generation of new clones. High-dimensional profiling techniques have led to an appreciation of the variety of states in AML. Apparently, aAVC-WT1 mobilized the full T cell repertoire for adaptive immune response. Our findings for the aAVC-WT1 therapy provide insights into the processes involved in establishing invigorated NK and T cells, resulting in a reduction of leukemic cells.

Our data show that aAVC therapy elicited T cell reinvigoration from T_PEX_ cells in the BM. Prior studies in solid tumors have shown that effector T and T_PEX_ cells can be recruited to and expand at tumor sites, and some of them undergo terminal exhaustion or become effective in response to checkpoint blockade.^43^^,^^44^^,^^45^ Reinvigoration of such cells has not been demonstrated in leukemia. We found that the FGFBP2^+^ T_EFF_ clusters were increased in all the patients who showed more than 50% reduction in the number of leukemic cells after the aAVC-WT1 therapy. The antitumor T cells in the BM were accompanied by the generation of T_PEX_ cells and FGFBP2^+^ T_EFF_ cells. In addition, a fraction of FGFBP2^+^ T_EFF_ cells reflect the pool of T_PEX_ cells, showing the ability to self-renew, and therefore, a kind of T_PEX_ cells persisted longer. On the contrary, the plasticity of T cells from T_PEX_ cells to functional ones was manifested in aAVC-WT1-treated patients with AML. In this study, we detected relatively high levels of *TCF7* in Inc clones at the pre-treatment stage, but their expression levels decreased after therapy (Figures S8C and S8D). TCF-1 (same as human TCF7) is a transcription factor that plays important roles in T cell development and differentiation. It was also reported that T_PEX_ cells are supported by TCF-1 to facilitate effector functions int the tumor microenvironment (TME).^46^^,^^47^^,^^48^ Inflammatory signals, such as interleukin-12 (IL-12) and type I IFN, together with TCR signals induce TCF-1 downregulation in primed CD8^+^ T cells.^48^ We showed that Inc clonotypes were derived mostly from pre-existing T cells after the administration of aAVC-WT1, and we assume that they might receive the signals of TCR and inflammatory cytokines by innate immunity. It is, therefore, conceivable that we detected high *TCF7* expression on increased T cell clones at the pre-treatment stage, but the expression decreased after therapy. Additionally, T_RM_ cells are a population of non-recirculating CD8^+^ T cells that reside permanently within peripheral tissues to mediate regional tumor surveillance. Tumor-localized T_RM_ cells in some cancers may better predict survival.^49^^,^^50^ Although we detected them in two patients, the significance of T_RM_ in AML needs further study.

Based on these clinical findings, along with our immunological observation, our data demonstrate that although patients with AML have impairment in both innate and adaptive immunity, activation of innate immunity using this novel platform led to DC-mediated activation of T cells, some of which were maintained long term. These studies, therefore, provide proof of concept in humans that an immunotherapy utilizing NKT-licensed DCs can lead to downstream activation of adaptive immunity *in vivo* and set the stage for further evaluation of this novel approach in AML, as well as in other cancers.

## Materials and methods

### Study design and procedure

Patients with RR-AML were enrolled in this phase 1 study. The patient characteristics and protocol are shown in Table 1 and Figure 1B, respectively. This was a single-center study of the 3 + 3 dose-escalation design (1 × 10^6^, 1 × 10^7^, and 1 × 10^8^/body), with the primary objective to establish the safety and biological activity of aAVC-WT1. The three patients in each cohort had not received any chemotherapy during more than 2 weeks before the therapy and had received aAVC-WT1 intravenously twice in a 4-week interval. All the adverse events that cannot be ruled out as being related to aAVC-WT1 are summarized in Table 2. All patients completed the dose-limiting toxicity (DLT) observation period, and no DLT occurred; the study ended with nine patients. In this case, there is an 86.6% chance that at least one adverse event, which occurs in 20% of patients, can be observed. Inclusion criteria required patients with relapsed or refractory AML are described in detail in the protocol synopsis (Table S1).

### Study drug and dosing

The cellular drug, aAVC-WT1, was manufactured at the CPC facility at the University of Tokyo by Lab for Immunotherapy, RIKEN-IMS. Safety was determined using the National Cancer Institute Criteria for Adverse Events v.4. Patients underwent a BM smear at enrollment every month. All patients underwent blood draws for immune assessment at baseline (pre-treatment) throughout the study and at end of study (EOS; post-treatment). Samples were transferred and analyzed at Lab for Immunotherapy at RIKEN-IMS.

### Treatment plan

The dose-escalation design required three groups of three patients enrolled sequentially: the first group with a dose of 1 × 10^6^ cells, the second with 1 × 10^7^ cells, and the third with 1 × 10^8^ cells. Progression to the higher dose would only take place if there were no DLTs. Patients were injected intravenously with each dose (1 × 10^6^, 1 × 10^7^, and 1 × 10^8^ cells/body) of aAVC-WT1 on days 0 and 28. Blood samples were obtained on pre (screening), on days 7, 14, 28, 42, and 56, and after 6 and 12 months. BM samples were obtained on pre (screening), on days 14, 28, 42, and 56, and after 6 and 12 months.

### Response criteria

Disease status was assessed using the modified criteria of the ELN;^29^ CR was defined as less than 5% of the leukemic blasts in the BM. Partial remission (PR) was defined as >50% decrease in BM blasts from baseline to 5%–25%. Stable disease (SD) was defined as absence of CR_MRD_, CR, CRi, PR, morphological leukemia-free state (MLFS), and progressive disease (PD). PD indicates >50% increase in marrow blasts over baseline (a minimum 15%-point increase is required in cases with <30% blasts at baseline or persistent marrow blast percentage >70% over at least 3 months). Leukemic blast cells were defined as CD45^lo^ population using flow cytometry (Figure S2A).

### Study approval

This trial was approved by The Institute of Medical Science, The University of Tokyo (IMSUT) (Tokyo, Japan) Institutional Review Board, the Pharmaceuticals and Medical Devices Agency, and registered at University Hospital Medical Information Network (UMIN) Center as UMIN000028083. All patients gave written informed consent before initiation of the study.

### Cell lines

HEK293 cells were purchased from American Type Culture Collection (Manassas, VA, USA) and serum-free cultured HEK293 (sfHEK293) cells were established by us.

### Preparation of aAVC-WT1

To load α-GalCer, sfHEK293 cells were cultured for 48 h in the presence of 500 ng/mL α-GalCer and then washed before electroporation with human *WT1* mRNA together with human *CD1d* mRNA. RNA electroporation of sfHEK293 cells was performed as follows. Briefly, cells were resuspended in OptiMEM (Thermo Fisher Scientific, Waltham, MA, USA) at a concentration of 5 × 10^7^ cells/mL. RNA was transferred to a 4-mm cuvette (Harvard Apparatus, Holliston, MA, USA), and the samples were pulsed in an ECM 830 Square Wave Electroporation System (Harvard Apparatus). Immediately after electroporation, the cells were transferred to a culture medium and cultured in the presence of 500 ng/mL α-GalCer (MEDINET, Tokyo, Japan). Transfected cells were analyzed using flow cytometry for CD1d and using western blot analysis for WT1. The quality, quantity, and safety evaluation for aAVC-WT1 were approved by the Pharmaceuticals and Medical Devices Agency (PMDA). For human use, aAVC-WT1 was irradiated (30 Gy) before the clinical trial.

### Human samples and preparation

PB and BM samples from 10 patients with AML who were enrolled in this phase 1 study were obtained from IMUST, The University of Tokyo. PBMCs were separated using Ficoll-Paque PLUS (GE Healthcare, Uppsala, Sweden) density centrifugation. PBMCs were washed twice with phosphate-buffered saline (PBS) (Nacalai Tesque, Kyoto, Japan) and stored in liquid nitrogen until use. Red blood cells were removed from BM samples by hemolysis with ACK buffer (Thermo Fisher Scientific). PB samples or buffy coat of healthy blood donors were obtained from our Institute or Tokyo Red Cross (Tokyo, Japan). PB samples from the patients with AML were obtained from the National Hospital Organization Kumamoto Medical Center (Kumamoto, Japan). All experiments were performed with authorization from the Institutional Review Board for Human Research at IMUST, The University of Tokyo, and RIKEN IMS. Written informed consent was obtained from all HDs and patients according to the Declaration of Helsinki. HLA-A24^+^ donors were selected using flow cytometry.

### Flow cytometry

Antibodies were purchased from BD Bioscience (Franklin Lakes, NJ, USA), BioLegend (San Diego, CA, USA), or Thermo Fisher Scientific are summarized in Table S4. A Fixable Aqua or Violet Dead Cell Stain Kit (Thermo Fisher Scientific) was used to eliminate dead cells. Intracellular staining for cytokines and transcription factors was performed using a BD intracellular cytokine staining kit (BD) and eBioscience Foxp3 Staining Buffer kit (Thermo Fisher Scientific), respectively. For analysis, a FACSCalibur, Canto II, or LSRFortessa X-20 instrument and CELLQuest, FACSDiva (BD Biosciences), or FlowJo software (v.10.3B2) (BD Biosciences) were used.

### ELISPOT assay

To evaluate human IFN-γ-secreting iNKT and NK cells, ELISPOT assays were performed by culturing with or without α-GalCer for 16 h, as previously described.^51^ Briefly, 96-well filtration plates (Merk Millipore, Burlington, MA, USA) were coated with mouse anti-human IFN-γ (10 μg/mL) (BD Biosciences). PBMCs (5 × 10^5^ cells/well) were incubated for 16 h with vehicle or α-GalCer (100 ng/mL) in R10. PMA (50 ng/mL) (Merk/SIGMA-Aldrich, St. Louis, MO, USA) and ionomycin (1 μg/mL) (Merk/SIGMA-Aldrich) were used as positive controls for T cell activation. After culture, the plates were washed and incubated with biotinylated anti-human IFN-γ (2 μg/mL) (BD Biosciences) for 2 h, and spots were developed with streptavidin-HRP (BD Biosciences) and stable DAB substrate (FALMA, Tokyo, Japan). IFN-γ SFCs were quantified by microscopy.

### Cellular immune response analysis of the WT1 synthetic peptide library

A library of 129 15-mer peptides spanning the WT1 protein with 11 overlapping residues was synthesized at the Proteomics Resource Center at the Rockefeller University (Table S3). The peptides were reconstituted in dimethyl sulfoxide at 50 mg/mL (as a stock solution), pooled as an all-peptide pool or into 10 subpools, and then stored at −80°C until use. PBMCs (3–5 × 10^6^ cells/well) were cultured with all peptide pools (each final concentration 2.5 μg/mL) in culture medium. On day 1, recombinant IL-2 (10 IU/mL; Roche Diagnostics Gmbh, Mannheim, Germany) and IL-7 (20 ng/mL; PeproTech, Rocky Hill, NJ, USA) were added. Additionally, recombinant IL-2 (50 IU/mL) and IL-7 (20 ng/mL) were added to the culture every other day. For 10- to 14-day expansion, PBMCs were restimulated with or without all mixed peptides in the presence of brefeldin A (50 μg/mL; Merk/SIGMA-Aldrich) and monensin (750 ng/mL; Merk/SIGMA-Aldrich) for 16 h and analyzed using flow cytometry. In some experiments, the cells were restimulated with each peptide subpool in the presence of brefeldin A and monensin or with all mixed peptides.

### Cell sorting

Human NK cells (CD3^−^CD56^+^) were sorted using a FACSAria (BD) and used for qPCR. Human T cells (CD45^hi^CD3^+^) were isolated and used for scRNA-seq and scTCR-seq.

### qPCR assay

For gene-specific reverse transcription (RT) and qPCR, oligonucleotide primer pairs and corresponding hydrolysis probes (UPL Probes) were designed at the Universal ProbeLibrary Assay Design Center on Roche’s web site. Primers are as follows: *IFNG* forward: 5′-GGCATTTTGAAGAATTGGAAAG-3′, *IFNG* reverse: 5′-TTTGGATGCTCTGGTCATCTT-3′, *PRF1* forward: 5′-CCGCTTCTCTATACGGGATTC-3′, *PRF1* reverse: 5′-GCAGCAGCAGGAGAAGGAT-3′, *GZMA* forward: 5′-TTAACCCTGTGATTGGAATGAAT-3′, *GZMA* reverse: 5′-AGGGCTTCCAGAATCTCCAT-3′, GAPDH forward: 5′-AGCCACATCGCTCAGACAC-3′, *GAPDH* reverse: 5′-GCCCAATACGACCAAATCC-3′; probes are as follows: *IFNG* (Roche Diagnostics 04686942001), *PRF1* (Roche Diagnostics 04688627001), *GZMA* (Roche Diagnostics 04685105001), *GAPDH* (Roche Diagnostics 04688589001). To obtain cDNA, NK cells were sorted using a FACSAria, and every 100 cells were directly lysed in 10 μL CellsDirect Reaction Buffer (Thermo Fisher Scientific) containing reverse transcriptase, Taq DNA polymerase, and a set of 200-nM primers. The lysates were then subjected to RT-PCR (30 min at 50°C followed by 2 min at 94°C) and subsequent pre-PCR (18 cycles of 30 s at 94°C and 4 min at 60°C) on a thermal cycler.

For qPCR, the reaction mixture contained a diluted aliquot of the above cDNA as a template, 500 nM of the same but single primer pair used for pre-PCR, a 250-nM UPL probe, and FastStart Universal Probe Master (ROX; Roche). The qPCR consisted of an initial activation step of 10 min at 95°C, followed by 40 cycles of 10 s at 95°C and 30 s at 60°C. qPCR was performed on a StepOne Plus (Thermo Fisher Scientific), and the average of duplicated results were analyzed by the comparative CT method with GAPDH expression as a reference.

### Bulk TCR-seq

Deep sequencing of the *TRB* gene was performed using the immunoSEQ platform (Adaptive Biotechnologies, South San Francisco, CA, USA) with genomic DNA extracted from PB with input amounts ranging from 5 to 16 ng. Only data from productive rearrangements were exported from the immunoSEQ Analyzer for further analysis.

### Preparation of scRNA-seq libraries

The scRNA-seq and scTCR-seq libraries were prepared using a 10× Single Cell Immune Profiling Solution Kit (10x Genomics, San Francisco, CA, USA) according to the manufacturer’s instructions. Briefly, fluorescence-activated cell sorting (FACS)-sorted cells were washed once with PBS containing 0.1% BSA and resuspended in PBS containing 0.1% BSA to a final concentration of 100–800 cells/μL, as determined using a hemocytometer. Cells were captured in droplets at a targeted cell recovery of 3,000 cells, resulting in estimated multiplet rates of 0.4%–5.4%. Following RT and cell barcoding in droplets, the emulsions were broken, and cDNA was purified using Dynabeads MyOne SILANE (Thermo Fisher Scienetific), followed by PCR amplification (98°C for 45 s; 14 cycles of 98°C for 20 s, 67°C for 30 s, 72°C for 1 min, and 72°C for 1 min). Amplified cDNA was then used for both five-gene expression library construction and TCR enrichment. For gene-expression library construction, 11.5–50 ng amplified cDNA was fragmented, end repaired, double-sided size selected with SPRIselect beads, PCR amplified with sample indexing primers (98°C for 45 s; 14–16 cycles of 98°C for 20 s, 54°C for 30 s, 72°C for 20 s; 72°C for 1 min), and double-sided size selected with SPRIselect beads (Beckman Coulter, Brea, CA, USA). For TCR library construction, TCR transcripts were enriched from 2 μL amplified cDNA by PCR (primer sets 1 and 2: 98°C for 45 s; 10 cycles of 98°C for 20 s, 67°C for 30 s, 72°C for 1 min, and 72°C for 1 min). Following TCR enrichment, 27.6–50.0 ng enriched PCR product was fragmented, end repaired, size selected with SPRIselect beads, PCR amplified with sample-indexing primers (98°C for 45 s; nine cycles of 98°C for 20 s, 54°C for 30 s, 72°C for 20 s, and 72°C for 1 min), and size selected with SPRIselect beads.

### Sequencing

The scRNA libraries were sequenced on a HiSeq 2500 to a minimum sequencing depth of 100,000 reads/cell using read lengths of 26 bp for read 1, 8 bp for the i7 index, and 98 bp for read 2. The single-cell TCR libraries were sequenced on an Illumina HiSeq 2500 (illumina, San Diego, CA, USA) to a minimum sequencing depth of 10,000 reads/cell using read lengths of 150 bp for read 1, 8 bp for i7 index, and 150 bp for read 2.

### scRNA-seq data processing

Raw gene-expression matrices were generated for each sample by the Cell Ranger (v.3.0.1) Pipeline coupled with human reference version GRCh38. The output filtered gene expression matrices were analyzed using R software (v.3.6.2) with the Seurat package (v.3.0.0.900). Briefly, genes expressed at a proportion >0.1% of the data and cells with >200 genes detected were selected for further analyses. Low-quality cells were removed if they met the following criteria: (1) <200 genes or (2) >10% unique molecular identifiers (UMIs) derived from the mitochondrial genome. On average, we obtained reads from 1,394 genes per cell (median: 1,323)*.* Clustering and gene expression were visualized with 10x Genomics Cell Browser v.2.0.0. Log_2_ fold change (FC) of assigned clusters was obtained via Loupe Cell Browser and used in the following analysis of differentially expressed genes within each cluster.

### Comparison of clusters with external single-cell gene signatures

We computed reference gene signatures from the scRNA-seq data of the datasets from Guo et al.^36^ Correlation matrix among clusters was obtained by corrcoef function in the Python numpy library. The average expression of signature genes in each cluster was drawn as a heatmap using the heatmap function in the Python seaborn library.

### Data processing of scTCR-seq libraries

TCR reads were aligned to the GRCh38 reference genome and consensus TCR annotation was performed using cellranger vdj (10x Genomics, v.3.0.1). TCR libraries were sequenced to a minimum depth of 10,000 reads per cell, with a final average of 34,123 reads per cell. On average, 30,078 reads mapped to either the *TRA* or *TRB* loci in each cell. TCR annotation was performed using the 10x cellranger vdj pipeline as described. In total, 88.4% of annotated T cells were assigned a TCR, and only 0.03% of cells not annotated as T cells were assigned a TCR. We detected an average of 1,021 unique clonotypes in each patient (range 23–3,522). Of 6,076 total clonotypes detected, an average of 1.84 cells were assigned to each clonotype, 1,556 clonotypes consisted of more than one cell, and clonotype sizes ranged from 1 to 44,672 cells. Using the Loupe browser, we categorized the CD8T cluster from the data of cloupe (gene expression) and vloupe (TCR) files in each sample and extracted information on CD8^+^ T cell clonotypes. Overall, 40% of T cells with TCR reads were assigned only TRB sequences, 6.8% of T cells with TCR reads were assigned TRA sequences, and 52% of T cells with TCR reads were assigned both TRB and TRA sequences. Therefore, T cells with TRB sequences were considered in the analysis because the number of T cells with both TRB and TRA sequences was quite limited.

### TCR repertoire stability

Assessment of TCR repertoire stability was performed using a Venn diagram approach (http://bioinformatics.psb.ugent.be/webtools/Venn/). The aaCDR3b sequence from BM CD8^+^ T cells in each patient at 3–5 time points was intersected using Venn diagrams. In addition, the aaCDR3b sequence from BM CD8^+^ T cells and PB bulk T cells at each time point in each patient were intersected using Venn diagrams.

### Statistics

All p values were calculated using the Tukey test. Log rank tests were used for survival calculations. Results with p *<*0.05 were considered statistically significant.

## Data availability

Data of scRNA-seq and sc-TCR-seq have been deposited at DDBJ (https://www.ddbj.nig.ac.jp/index.html) under NBDC: JGAS000555.

## Supplemental information

Document S1. Table S2 and Figures S1–S8

Table S1. Protocol synopsis

Table S3. WT1 peptide library

Table S4. Antibody panel for flow cytometry

Document S2. Article plus supplemental information
