# Supplementary material for: Reinvigoration of innate and adaptive immunity via therapeutic cellular vaccine for patients with AML
Source: Mol Ther Oncolytics. 2022 Sep 23;27:315–32. doi: 10.1016/j.omto.2022.09.001 (PMC12447268; doi:10.1016/j.omto.2022.09.001)
Supplement: Document S1. Table S2 and Figures S1–S8 [file mmc1.pdf]

OMTO, Volume *27*

Supplemental information

Reinvigoration of innate and adaptive immunity via therapeutic cellular vaccine for patients with AML

Shin-ichiro Fujii, Toyotaka Kawamata, Kanako Shimizu, Jun Nakabayashi, Satoru Yamasaki, Tomonori Iyoda, Jun Shinga, Hiroshi Nakazato, An Sanpei, Masami Kawamura, Shogo Ueda, Jan Dörrie, Svetlana Mojsov, Madhav V. Dhodapkar, Michihiro Hidaka, Masanori Nojima, Fumitaka Nagamura, Shigemi Yoshida, Toshio Goto, and Arinobu Tojo

Table S1.Protocol Synopsis

Table S2. T cell response.

Cohort	Pt. No.	Sample	CD4$^+$ T post*/pre (fold)	CD8$^+$ T post*/pre (fold)
Cohort-1	A001	PB**	222.42/93.53 (2.38)	507.18/187.65 (2.70)
	A002		111.92/105.31 (1.06)	23.13/21.36 (1.08)
	A003		102.72/150.02 (0.68)	83.78/130.50 (0.64)
	Means ±SEM (fold)		**1.38±0.42**	**1.48±0.51**
	A001	BM***	22.5%/17.8% (1.26)	48.9%/ 49.0% (1.00)
	A002		43.0%/42.9% (1.00)	12.1%/ 13.2% (0.92)
	A003		41.0%/33.7% (1.22)	47.1%/ 45.9% (1.03)
	Means ±SEM (fold)		**1.16±0.07**	**0.98±0.03**
Cohort-2	A004	PB**	271.13/229.59 (1.18)	57.29/40.60 (1.41)
	A005		453.80/377.40 (1.20)	303.85/268.03 (1.13)
	A006		373.12/237.02 (1.57)	249.19/164.71 (1.51)
	Means ±SEM (fold)		**1.32±0.10**	**1.35±0.09**
	A004	BM***	29.5%/ 31.5% (0.94)	12.1%/ 11.9% (1.01)
	A005		37.3%/ 24.6% (1.52)	31.9%/ 29.2% (1.09)
	A006		29.6%/ 29.8% (1.00)	44.0%/ 30.4% (1.45)
	Means ±SEM (fold)		**1.15±0.15**	**1.18±0.11**
Cohort-3	A007	PB**	539.37/302.67 (1.78)	146.87/129.14 (1.14)
	A008		174.39/493.79 (0.35)	102.72/415.30 (0.25)
	A010		168.69/196.43 (0.86)	91.34/46.92 (1.95)
	Means ±SEM (fold)		**1.00±034**	**1.11±0.40**
	A007	BM***	34.1%/ 30.4% (1.12)	19.4%/ 18.8% (1.03)
	A008		27.6%/ 22.0% (1.25)	31.9%/ 28.2% (1.13)
	A010		N.D.	N.D.
	Means (fold)		**1.19**	**1.08**

*best response in all time course; ** (cells /μL) in PB, ***relative frequency (%) in BM CD45hi cells, N.D. not done

Table S3. WT1 peptide library

Table S4. Antibody panel for flow cytometry

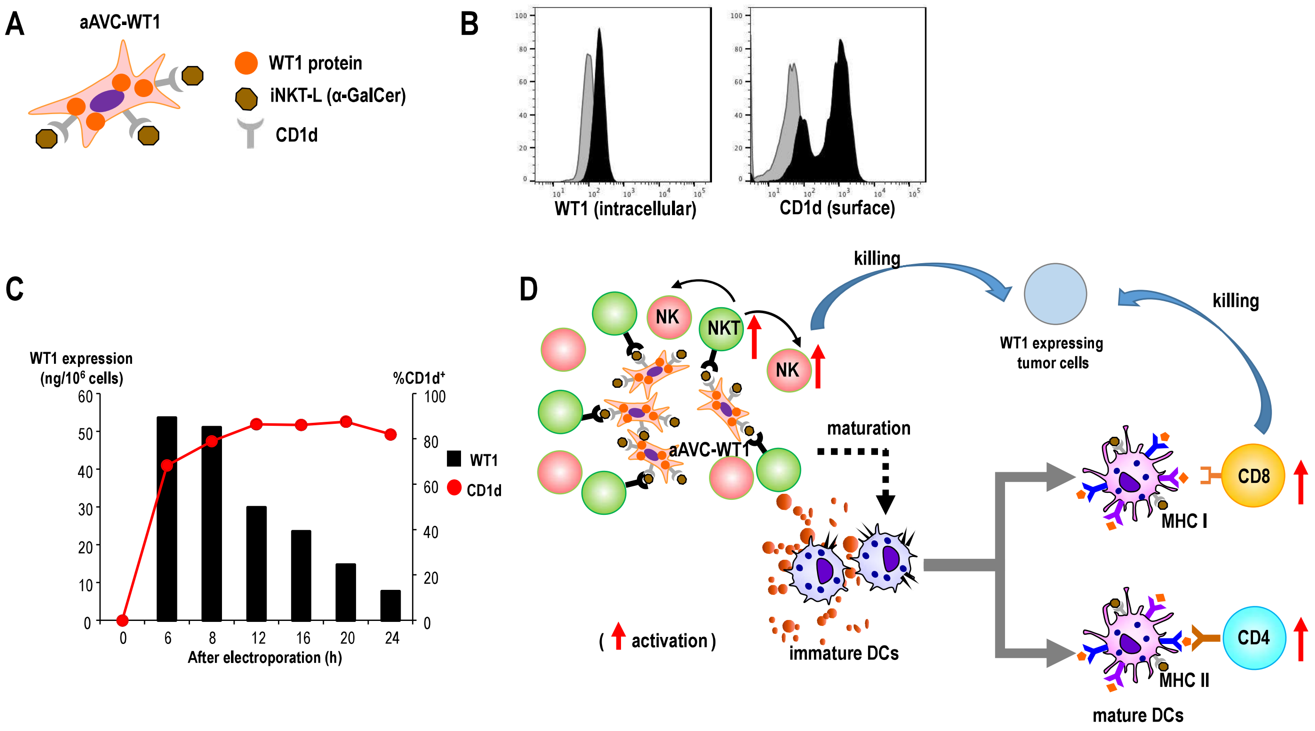

Figure S1. Immunological mechanism of aAVC-WT1 treatment.

(A) aAVC-WT1 expresses the CD1d/α-GalCer complex on the surface and WT1 protein intracellularly. **(B)** Expression of intracellular WT1 and surface CD1d of aAVC-WT1 assessed using flow cytometry analysis (black, WT1 or CD1d; gray, isotype). **(C)** The kinetics of WT1 and CD1d expression after mRNA electroporation in sfHEK293 cells. WT1 expression and CD1d expression was determined using western blot and flow cytometry analyses, respectively. (D) AVC-WT1 can activate iNKT cells directly and NK cells via iNKT cell activation indirectly. Although aAVC-WT1 cells were killed by iNKT and NK cells, the debris of aAVC-WT1 is efficiently captured by DCs *in situ.* Subsequently, *in vivo* DCs undergo maturation. Finally, antigen-captured DCs can present WT1 antigens to $CD4^+$ T cells and exhibit cross-presentation to $CD8^+$ T cells.

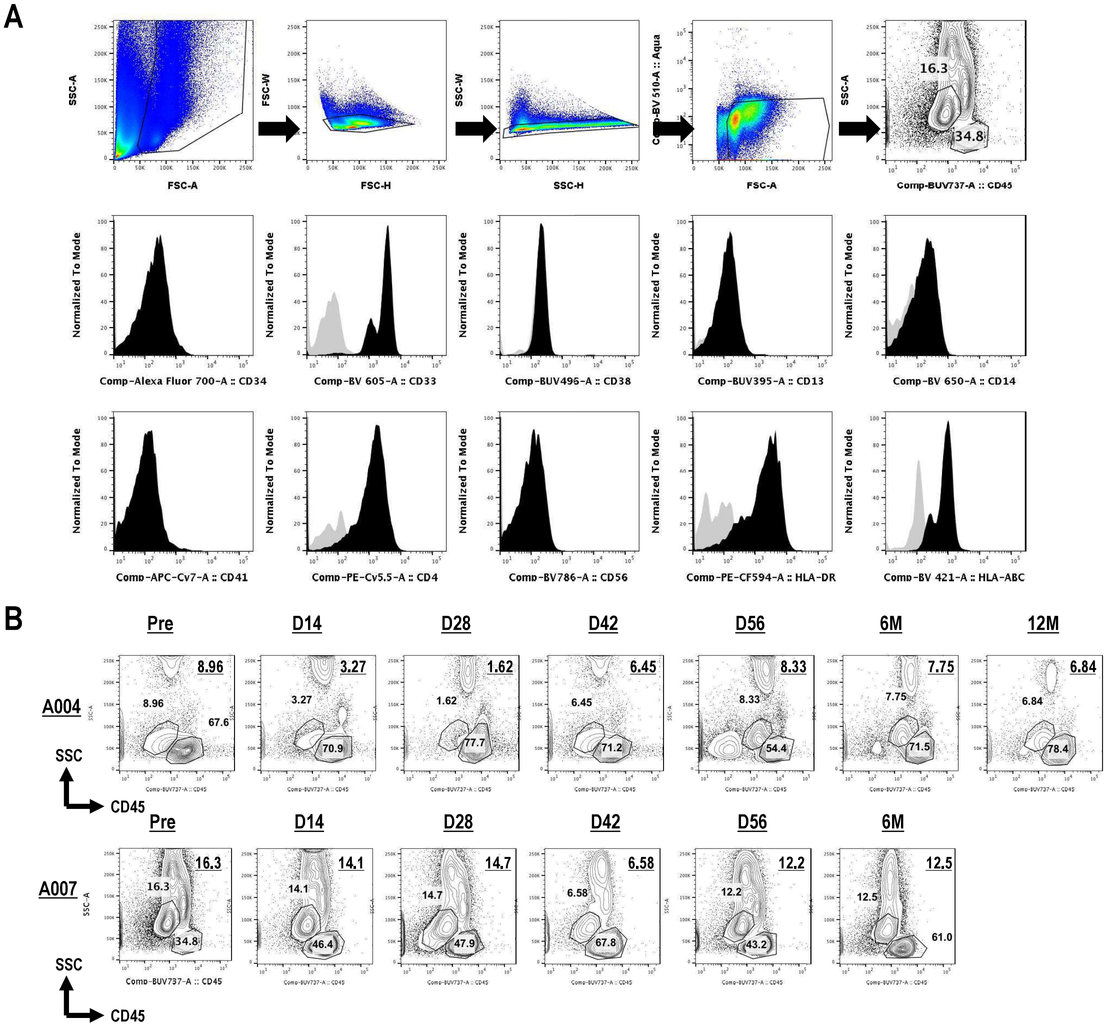

Figure S2. Gating strategy and monitoring of leukemic blast in BM by flow cytometry.
(A) Gating strategy for leukemic blasts. The percentage of leukemic blasts (CD45^{lo}) was determined after exclusion of debris, cell doublets, and dead cells, and the expression of indicated leukemic cell markers was confirmed. **(B)** Percentage of leukemic blasts (CD45^{lo}) in the BM, as evaluated using flow cytometry, in the representative patients, A004 (upper) and A007 (lower).

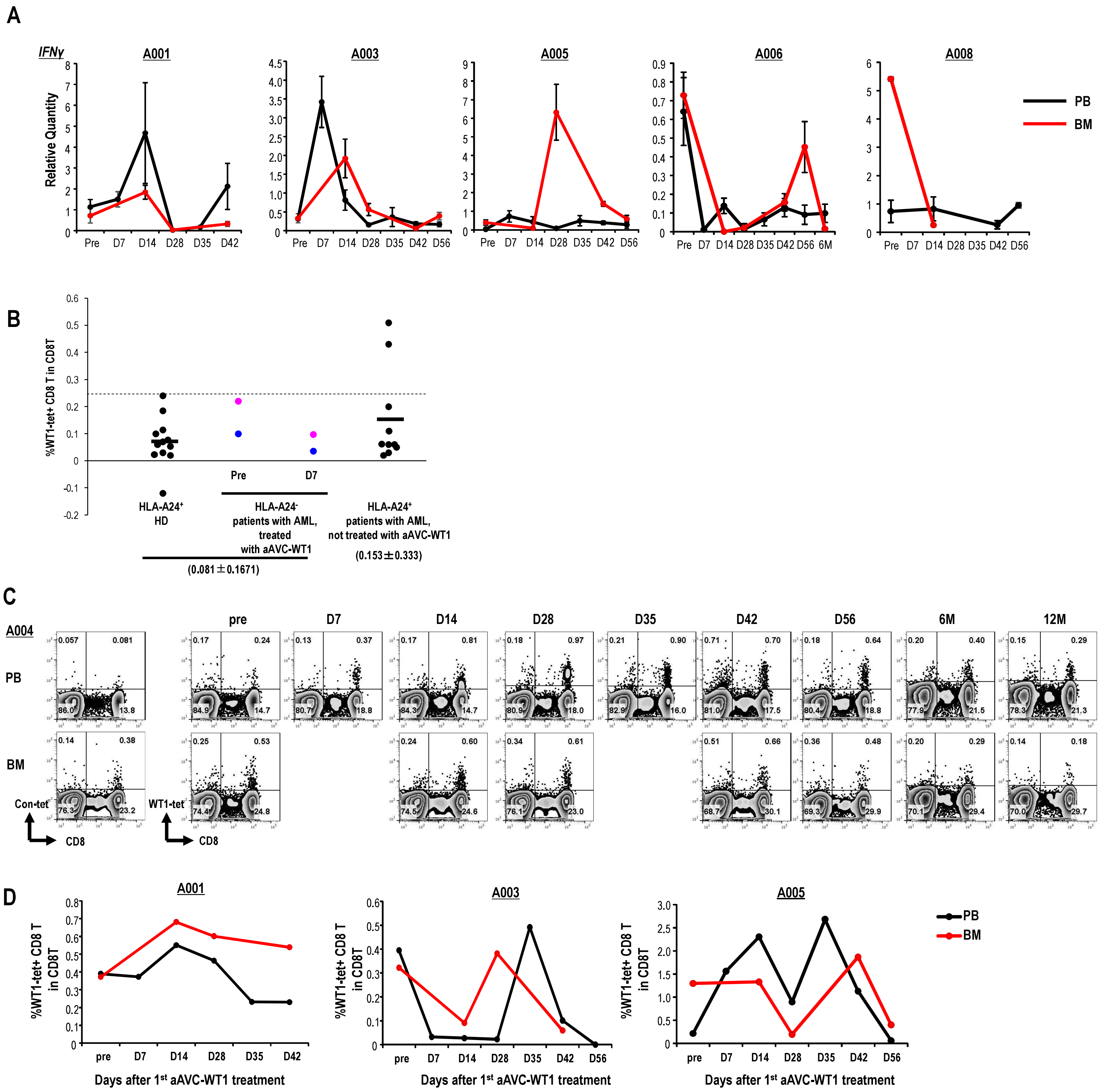

Figure S3. Innate immunity and WT1 specific T cell response in aAVC-WT1-treated patients with RR-AML. (A) Expression of *IFNG* in NK cells sorted from the PB (Black) and BM (Red), as determined by qPCR in A001, A003, A005, A006 and A008. **(B)** The background of WT1/HLA-A24:02 tetramer assay. PBMCs from HLA-A24$^+$ healthy donors (HD) (n=12), HLA-A24$^+$ patients with AML who were not treated with aAVC-WT1 (n=10) and HLA-A24$^-$ patients with AML who were treated with aAVC-WT1 (A007 and A008) were stained with WT1/HLA-A24:02-tetramer and HIV peptide/HLA-A24:02 tetramer (negative control) and analyzed by flow cytometry. The tetramer-positive frequency was calculated by subtracting the frequency of the negative. Black bar represents the mean. Numbers indicate mean ± 2SD. **(C)** WT1 peptide/HLA-A24:02 tetramer assay of primary PB (upper) and BM (lower) in representative patient A004, as determined using flow cytometry. Data were gated on CD45$^+$CD3$^+$ T cells. The left column shows negative control tetramer (HIV peptide/HLA-A2402 tetramer) staining. **(D)** Kinetics of the frequency of HLA-A24:02 WT1-tet$^+$CD8$^+$ T cells in CD8$^+$ T cells from primary PBMCs (black) and BMMNCs (red) in patients A001, A003, and A005. The tetramer-positive frequency was calculated by subtracting the frequency of the negative control.

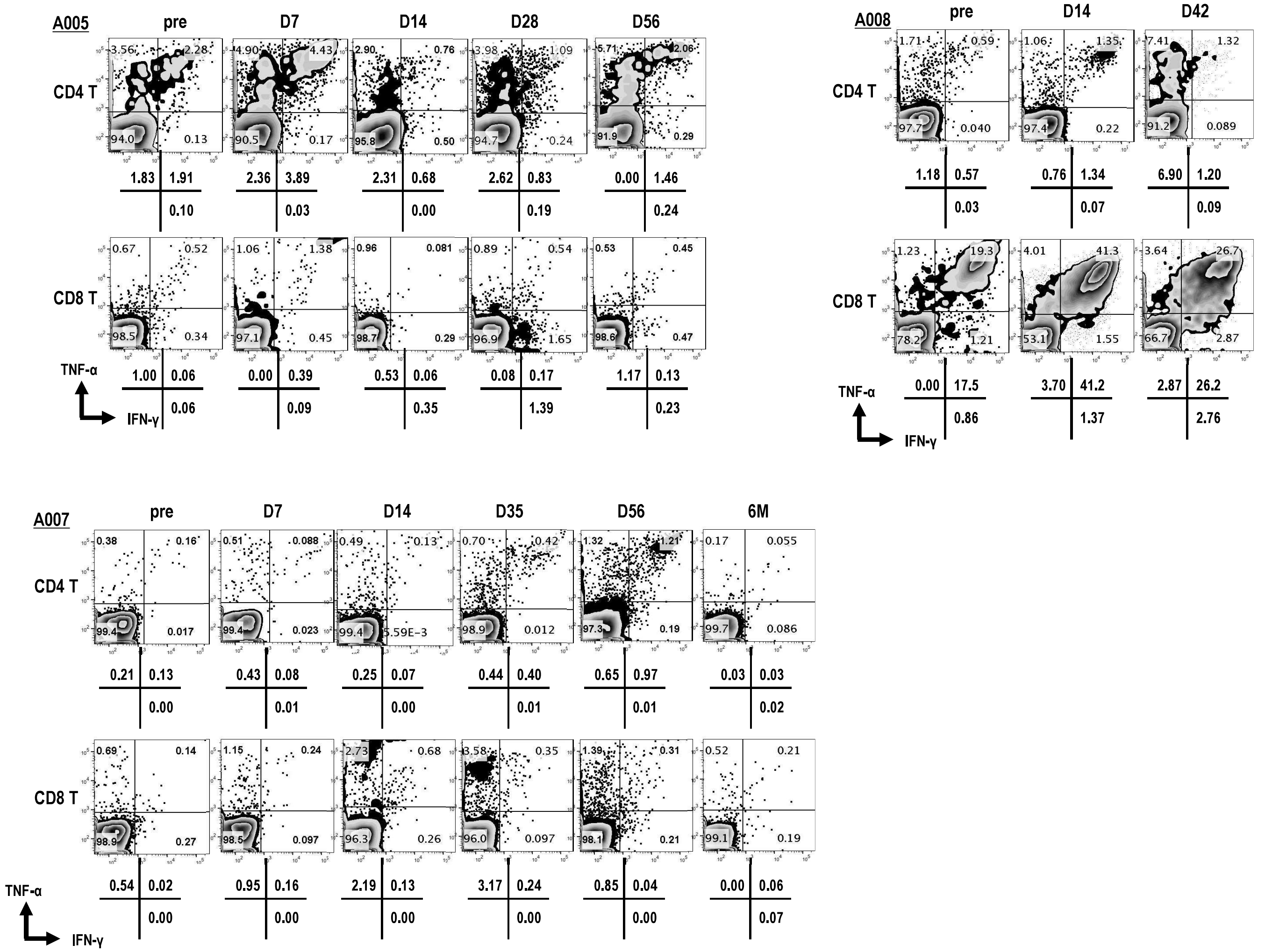

Figure S4. The responsiveness of T cells to the WT1 peptide library

WT1-specific $CD4^+$ T and $CD8^+$ T cell responses in PB using the WT1 peptide library. Intracellular IFN-γ and TNF-α production by $CD4^+$ T and $CD8^+$ T cells was determined using flow cytometry. Representative flow cytometry data are shown for patient A005, A007 and A008. Data on gating of $CD3^+CD4^+$ for $CD4^+$ T cells (upper) and $CD3^+CD8^+$ for $CD8^+$ T cells (lower) are shown. The numbers indicate the frequency of three subsets (TNF-α^+IFN-γ^-, TNF-α^-IFN-γ^+, and TNF-α^+IFN-γ^+) by subtracting the frequency of non-stimulated control.

A

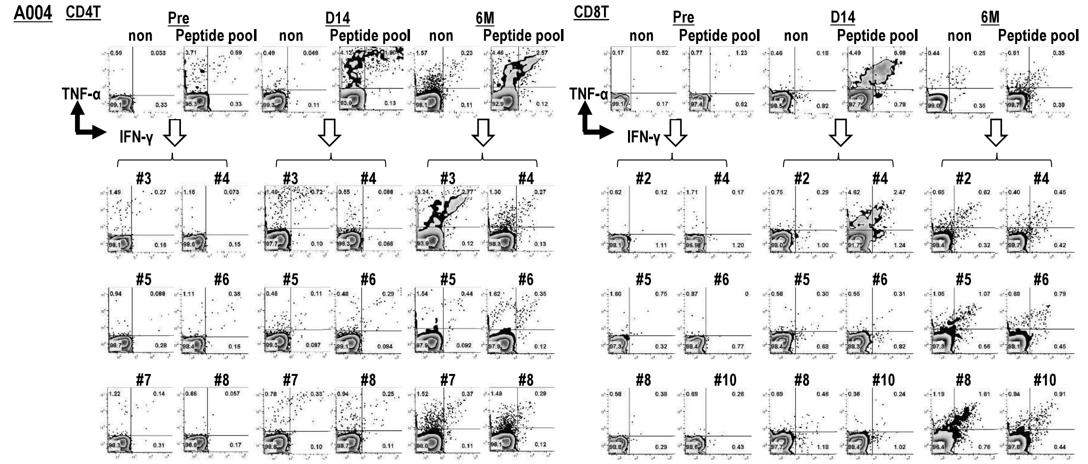

B

CD4T

TNF-α+IFN-γ-

	#1	#2	#3	#4	#5	#6	#7	#8	#9	#10
Pre			+				+			
D7	+		+++				+			
D14	+		++				+	+		
6M			++							
12M			+				+			

TNF-α-IFN-γ+

	#1	#2	#3	#4	#5	#6	#7	#8	#9	#10
Pre										
D7										
D14										
6M										
12M										

TNF-α+IFN-γ+

	#1	#2	#3	#4	#5	#6	#7	#8	#9	#10
Pre						+				
D7	+		+		+	+	+	+		
D14			+			+	+	+		
6M	+		++		+					
12M										

CD8T

TNF-α+IFN-γ-

	#1	#2	#3	#4	#5	#6	#7	#8	#9	#10
Pre	+			++	+	+			+	+
D7		+		+	+	+		+		
D14		+	+	+++				+		
6M	+	+	+		++	+		++		+
12M	+		+		+		+	+		

TNF-α-IFN-γ+

	#1	#2	#3	#4	#5	#6	#7	#8	#9	#10
Pre	+	+		+	+	+	+	+		+
D7				+	+	+		+		
D14				+				+		+
6M					+			+		
12M			+							

TNF-α+IFN-γ+

	#1	#2	#3	#4	#5	#6	#7	#8	#9	#10
Pre			+							
D7		+	+	+	+		+	+	+	+
D14			+	++				+		
6M	+	+	+	+	+	+	+	++	+	+
12M			+				+			

C

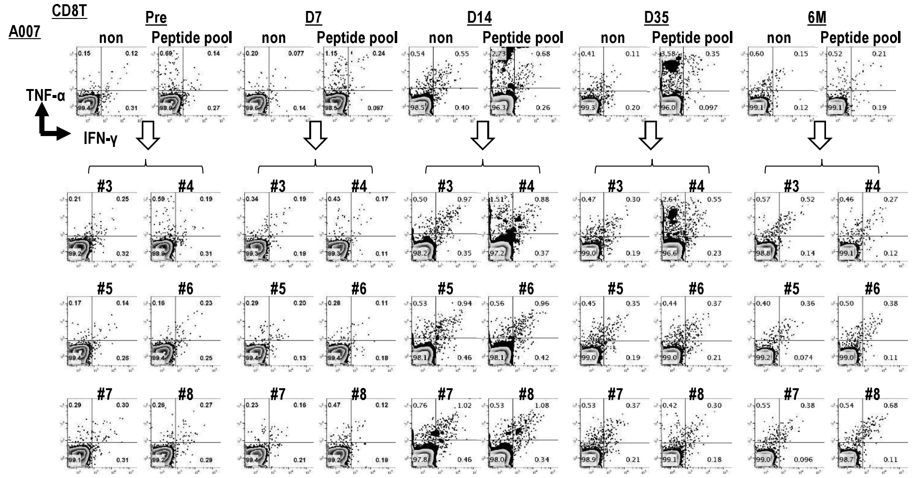

D

CD8T

TNF-α+IFN-γ-

	#1	#2	#3	#4	#5	#6	#7	#8	#9	#10
Pre				+						
D7				+				+		
D14				+			+			+
D35				++						
6M										

TNF-α-IFN-γ+

	#1	#2	#3	#4	#5	#6	#7	#8	#9	#10
Pre										
D7										
D14										
D35										
6M										

TNF-α+IFN-γ+

	#1	#2	#3	#4	#5	#6	#7	#8	#9	#10
Pre										
D7										
D14	+		+	+	+	+	+	+		+
D35		+		+	+	+	+			
6M			+		+	+	+	+		

Figure S5. The responsiveness of T cells to subpools of the WT1 peptide library

(A, C) As in Fig.4**B**, but the responsiveness of CD4$^+$ and CD8$^+$ T cells to subpools of the WT1 peptide library in A004 **(A)** and the responsiveness of CD8$^+$ T cells to subpools of the WT1 peptide library in A007 **(C)**. **(B, D).** The frequency of the three subsets of T cells (TNF-α$^+$IFN-γ$^-$, TNF-α$^-$IFN-γ$^+$, and TNF-α$^+$IFN-γ$^+$) in A004 (B) and A007 (D) was calculated by subtracting the frequency of non-stimulated control. (+, 0.2–1.0%; ++, 1.0–3.0%; +++, > 3.0%).

A

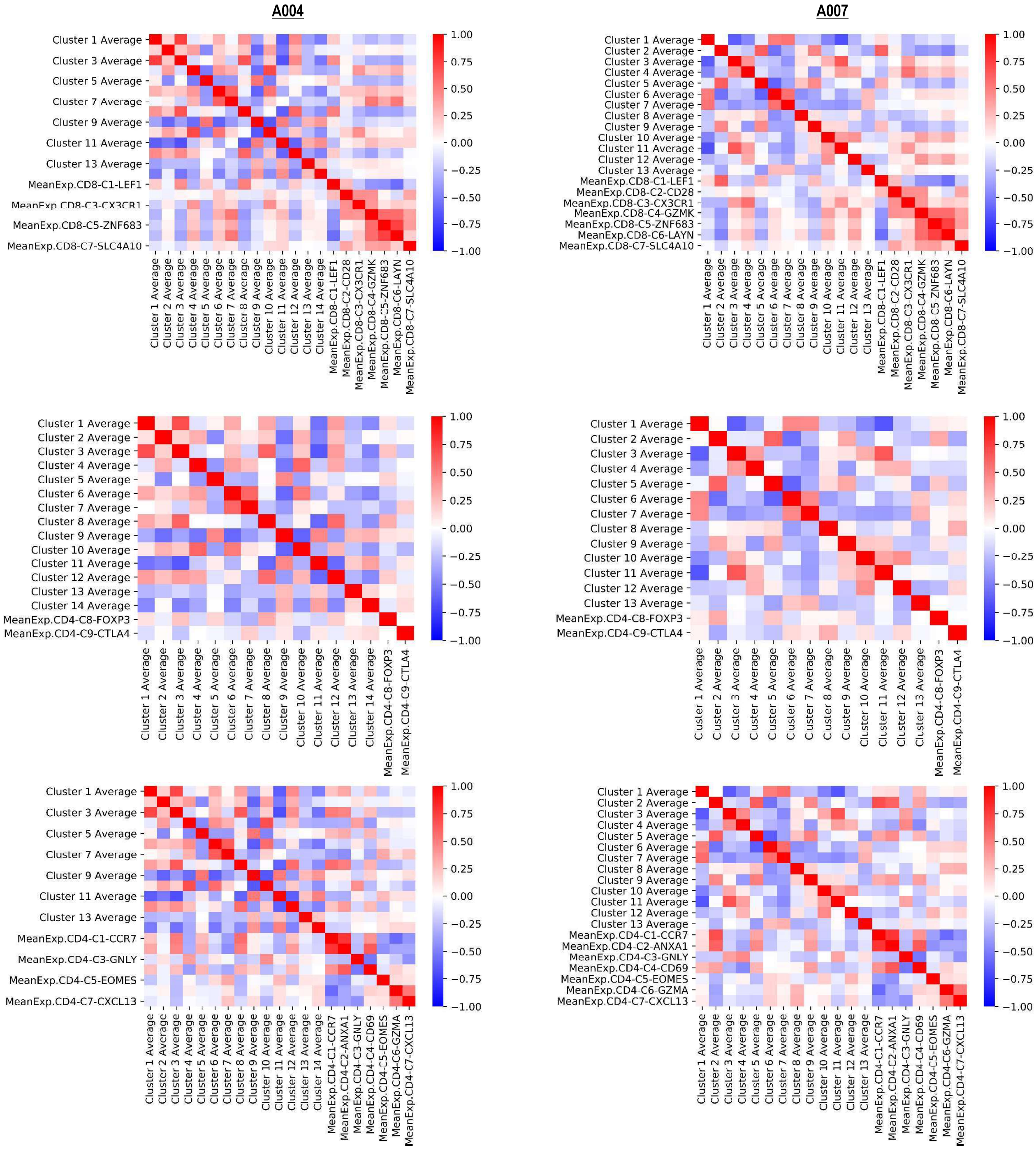
A004
A007
Cluster 1 Average
Cluster 3 Average
Cluster 5 Average
Cluster 7 Average
Cluster 9 Average
Cluster 11 Average
Cluster 13 Average
MeanExp.CD8-C1-LEF1
MeanExp.CD8-C3-CX3CR1
MeanExp.CD8-C5-ZNF683
MeanExp.CD8-C7-SLC4A10
1.00
0.75
0.50
0.25
0.00
−0.25
−0.50
−0.75
−1.00
Cluster 2 Average
Cluster 4 Average
Cluster 6 Average
Cluster 8 Average
Cluster 10 Average
Cluster 12 Average
Cluster 14 Average
MeanExp.CD8-C2-CD28
MeanExp.CD8-C4-GZMK
MeanExp.CD8-C6-LAYN
MeanExp.CD4-C8-FOXP3
MeanExp.CD4-C9-CTLA4
MeanExp.CD4-C1-CCR7
MeanExp.CD4-C2-ANXA1
MeanExp.CD4-C3-GNLY
MeanExp.CD4-C4-CD69
MeanExp.CD4-C5-EOMES
MeanExp.CD4-C6-GZMA
MeanExp.CD4-C7-CXCL13

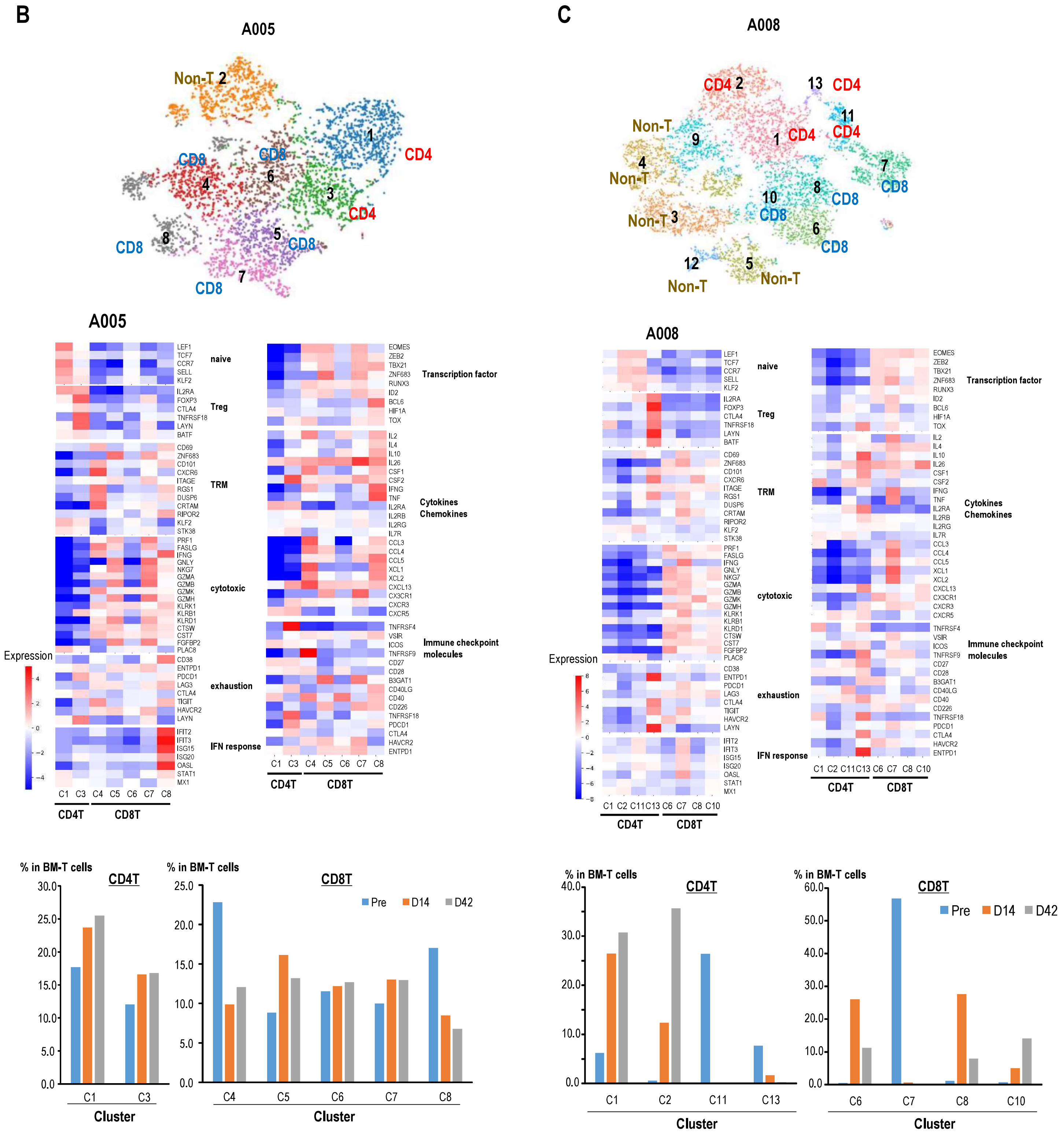

Figure S6. T-cell heterogeneity in BM.
(A) Characterization of T-cell clusters. Heatmaps show cross-labelling of T-cell clusters (columns) to reference gene signatures (rows) taken from the analysis by Guo et al. with intensities indicating normalized frequency. (**B, C**) Characterization of BM-CD4⁺ and CD8⁺ T-cell clusters in two patients (A005 and A008). The tSNE projection of single T cells showed the formation of 8 and 13 clusters in A005 **(B)** and A008 **(C)**, respectively. Each dot corresponds to a single cell, colored according to the cell cluster (upper). Average $\log_2$ fold change through all time points of selected T-cell function-associated genes in each cell cluster (middle). Kinetics of the frequency of each cluster (lower).

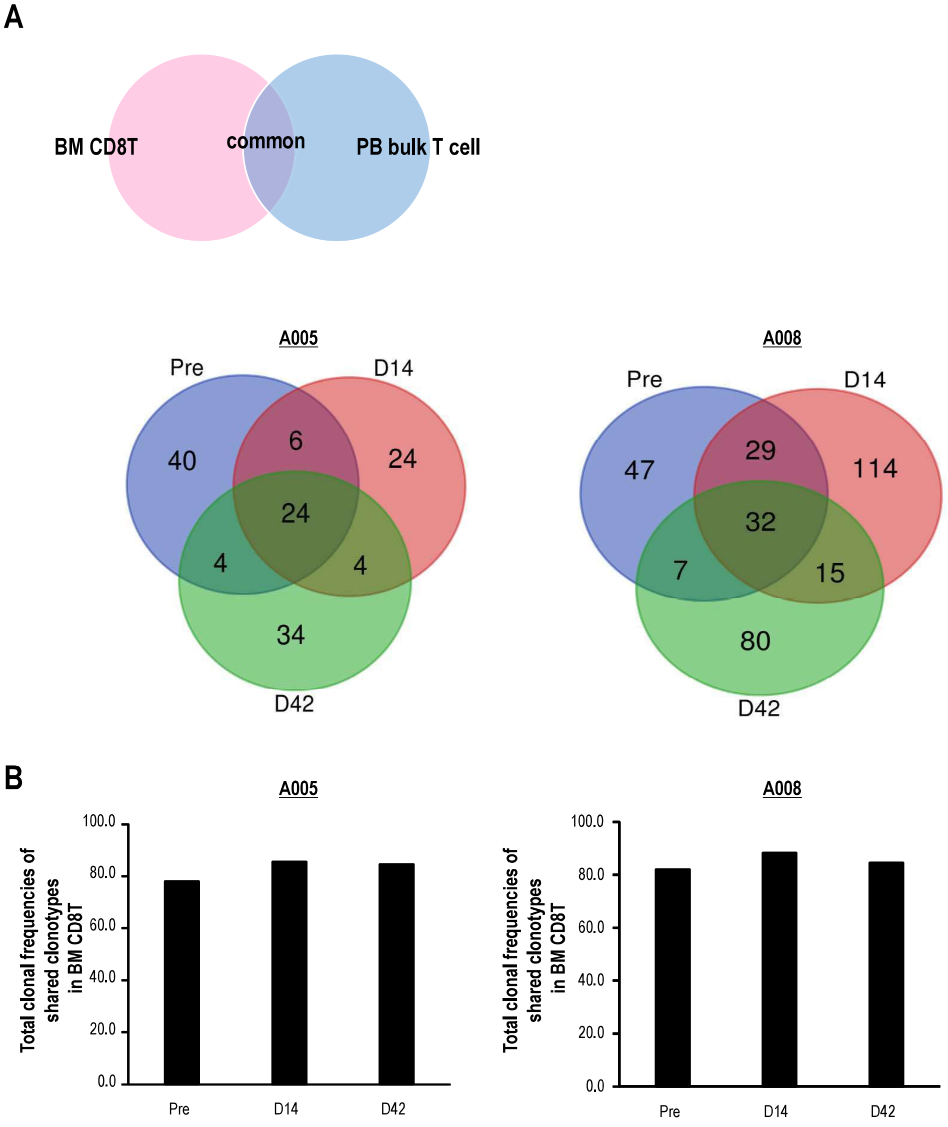

Figure S7. TCR repertoire analysis in PB and BM.
As shown in **Fig. 5C,** overlap between TRB clones in PB and BM T cells detected by bulk TCR**-**seq for PB T cells and scTCR**-**seq for BM T cells in patients A005 and A008. **(A)** Venn diagram showing the sharing of TCR clonotypes in the BM and PB (A005 and A008). **(B)** The total clonal frequencies of shared TCR clones in BM**-**CD8^{+} T cells in A005 and A008.

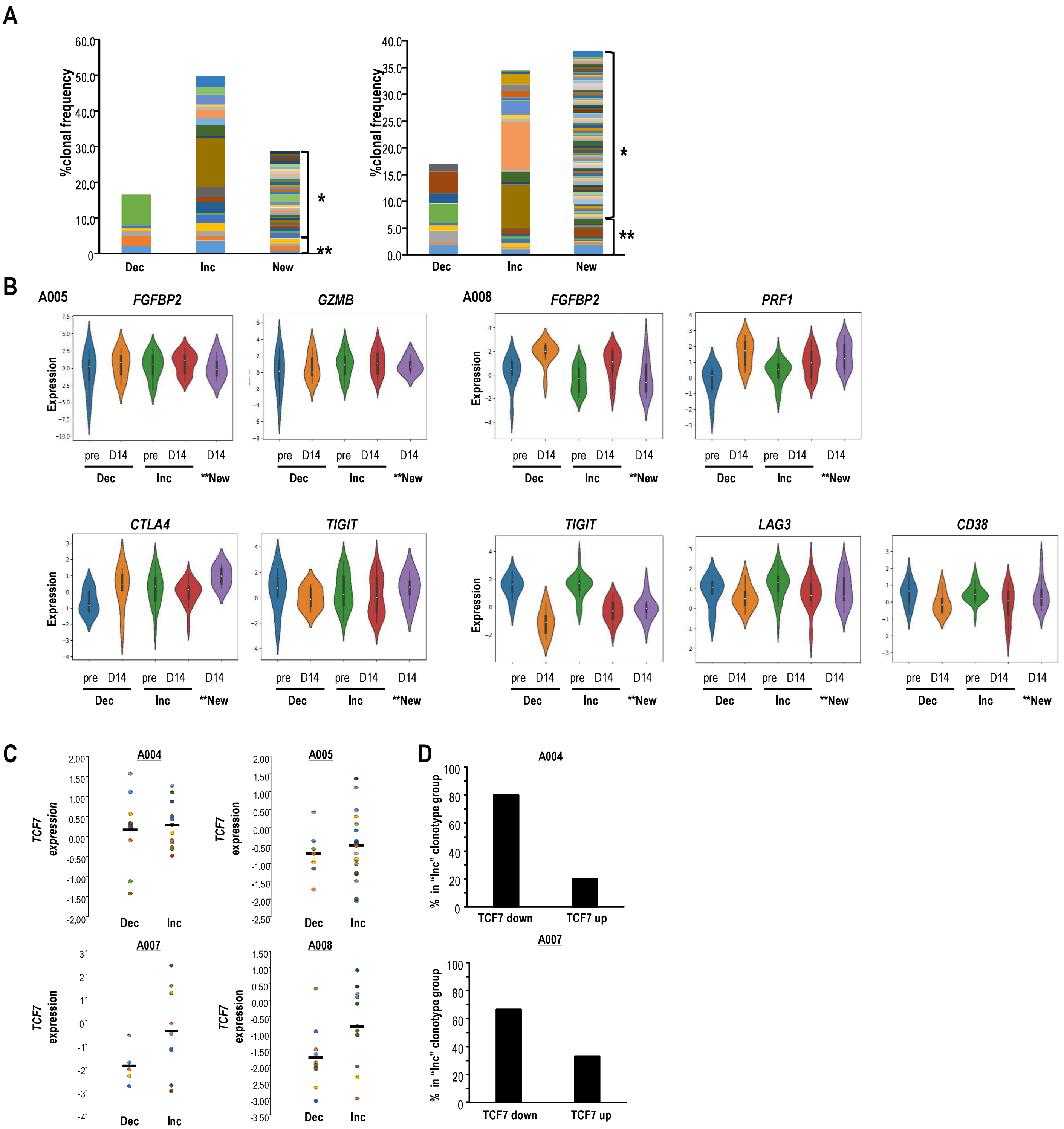

Figure S8. Kinetics of CD8^{+} T clonotypes in BM.
(A) As shown in **Fig. 5A, c**lone frequencies of "increased" clones (Inc), "decreased" clones (Dec), and "new" clones (New) in BM-CD8^{+} T cells on day 14 in patients A005 and A008. (*indicates the transient clones only at day 14, whereas ** indicates stable clones remaining from day14 to day 42.) (**B)** Expression of a given gene in the Inc, Dec, and New groups at pretreatment and on day 14 in patients A005 and A008. **(C)** Expression of *TCF7* at pretreatment in the Inc and Dec groups in patients A004, A005, A007, and A008. **(D)** Compared to pretreatment, T-cell clonotypes on day 14 after aAVC-WT1 therapy are assessed by down- or up-regulation of *TCF7* expression in patients A004 and A007. Percentages in Inc groups indicate the ratio of these two groups out of all clonotypes.
